# Metabolic imaging in living plants: A promising field for chemical exchange saturation transfer (CEST) MRI

**DOI:** 10.1126/sciadv.adq4424

**Published:** 2024-09-18

**Authors:** Simon Mayer, Hardy Rolletschek, Volodymyr Radchuk, Steffen Wagner, Stefan Ortleb, Andre Gündel, Klaus J. Dehmer, Fabian T. Gutjahr, Peter M. Jakob, Ljudmilla Borisjuk

**Affiliations:** ^1^ Leibniz Institute of Plant Genetics and Crop Plant Research (IPK), Corrensstrasse 3, 06466 Seeland-Gatersleben, Germany.; ^2^ Institute of Experimental Physics 5, University of Würzburg, Am Hubland, 97074 Würzburg, Germany.; ^3^ Department of Ecology, Environment and Plant Sciences, Stockholm University, SE-106 91 Stockholm, Sweden.

## Abstract

Magnetic resonance imaging (MRI) is a versatile technique in the biomedical field, but its application to the study of plant metabolism in vivo remains challenging because of magnetic susceptibility problems. In this study, we report the establishment of chemical exchange saturation transfer (CEST) for plant MRI. This method enables noninvasive access to the metabolism of sugars and amino acids in complex sink organs (seeds, fruits, taproots, and tubers) of major crops (maize, barley, pea, potato, sugar beet, and sugarcane). Because of its high signal detection sensitivity and low susceptibility to magnetic field inhomogeneities, CEST analyzes heterogeneous botanical samples inaccessible to conventional magnetic resonance spectroscopy. The approach provides unprecedented insight into the dynamics and distribution of sugars and amino acids in intact, living plant tissue. The method is validated by chemical shift imaging, infrared microscopy, chromatography, and mass spectrometry. CEST is a versatile and promising tool for studying plant metabolism in vivo, with many applications in plant science and crop improvement.

## Teaser

CEST MRI presents a promising tool for noninvasive monitoring of sugars and amino acids in plants.

## INTRODUCTION

The “omics” technologies—genomics, transcriptomics, proteomics, and metabolomics—are at the forefront of discovery in modern plant science and systems biology (*1*–*3*). In contrast to the more “static” genome, the metabolome and the products measured within it are very dynamic and are regulated both spatially and temporally. In the biomedical field, the most powerful technological platform allowing for in vivo metabolic diagnostic and functional studies is nuclear magnetic resonance (NMR) imaging or magnetic resonance imaging (MRI) (*4*). In plant science, a similar perspective has been desired but still has not been explored (*5*). Common ^1^H NMR imaging of biological tissue relies on signals primarily originating from water or lipid protons (*6*). As the concentration of metabolite protons is at least three orders of magnitude lower than that of water, the in vivo detection of metabolites requires effective suppression of the water signal (*7*, *8*). The success of this strategy largely relies on achieving a homogeneous magnetic field (*9*–*12*), which is essential for spectroscopic methods such as chemical shift imaging (CSI) (*13*). However, this can be challenging to achieve because of the unique structural peculiarities of plant tissues (*14*). For example, intercellular air spaces result in magnetic susceptibility artifacts (*15*). Another option is to label the metabolites, as demonstrated by ^13^C/^1^H NMR imaging, but the experiments are time-consuming and demanding (*9*, *15*–*17*). The need for noninvasive technologies is growing to address important questions in plant biology (*5*, *18*), particularly mechanisms of metabolite partitioning and sink-source relationships underlying plant growth and propagation (*19*–*21*).

Chemical exchange saturation transfer (CEST), a different approach that is used in the biomedical field, could offer an alternative solution. In CEST, magnetization is transferred from other molecules to water molecules, so that the saturation effect (i.e., signal reduction) that was originally on the targeted species can instead be observed on water. In that way, CEST enables the detection of various metabolites based on their ability to exchange protons with water thereby providing an additional MRI contrast (*22*, *23*). Plants contain numerous molecules, particularly amino acids with amine groups and saccharides with mobile protons of hydroxyl groups, that have this property, and initial attempts have been made to use CEST in this context (*24*, *25*). In our work, we aimed to unlock the potential of CEST for imaging metabolites in living plants, focusing on those abundant metabolite classes (saccharides and amino acids) whose availability in the sink organs (such as seed, fruits, roots, and stem) is critical for plant growth and crop yield.

## RESULTS

### Experimental design and application of CEST on plants

**Fig. 1.**
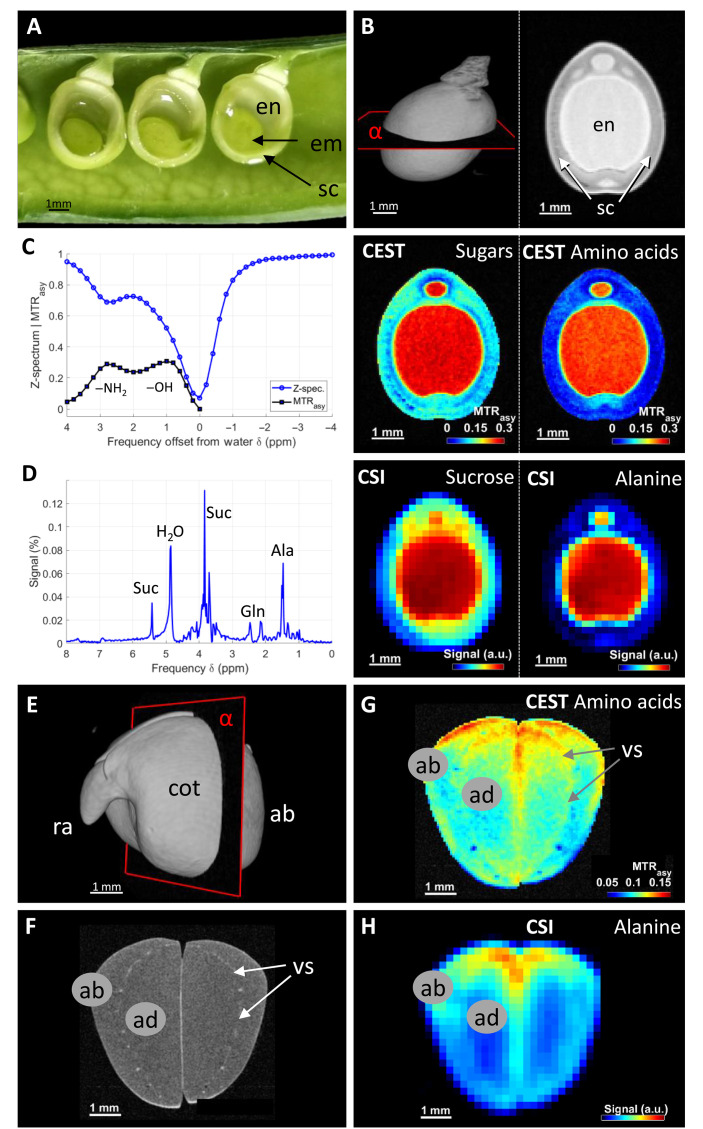
Metabolite imaging using CEST and CSI in developing pea seeds. (**A**) Photographic image of dissected seeds showing liquid endosperm (en), embryo (em), and seed coat (sc). (**B**) Three-dimensional (3D) MRI model of intact seeds at the early developmental stage and the virtual slice (α) used for measurements in (C) and (D). (**C**) A representative CEST spectrum (left) and CEST maps of seeds (right). (**D**) A representative CSI spectrum (left) (normalized to the water signal measured by CSI without water suppression) and corresponding metabolite images by integration of the sucrose and alanine peaks (right). (**E**) MRI 3D model of pea embryo at late developmental stage and orientation of the measured slice (α) in (F) to (H). (**F**) Structural MRI of the measured slice. (**G** and **H**) Visualization of amino acid distribution measured by CEST and CSI. Sugar distribution is shown in fig. S7. ab, abaxial parenchyma; ad, adaxial parenchyma; cot, cotyledon; ra, radicle; Suc, sucrose; Ala, alanine; Gln, glutamine; vs, vasculature; a.u., arbitrary units.

In higher plants, seed development involves three genetically and functionally distinct compartments (embryo, endosperm, and seed coat) that compete for assimilates supply to realize growth potential (*19*, *26*). Seeds of pea (*Pisum sativum* L.) were subjected to NMR analysis at distinct stages of development. During the early stages of development, the seed consisted of a large portion of liquid endosperm (Fig. 1A), which plays a nurturing role for the embryo (*10*) and was clearly visible in the MRI of an intact seed (Fig. 1B). Chromatography detected high levels of metabolites in dissected endosperm at this stage, with sucrose (Suc), alanine (Ala), and glutamine (Gln) as the main compounds (fig. S1, A and B). We attempted to noninvasively visualize the content of sugars and amino acids in the endosperm of the intact seed using both classical CSI and CEST approaches. In a CEST experiment, the exchange of metabolite protons with water protons is measured. This is achieved by frequency-selective saturation pulses that saturate (reduce) the magnetization of the exchanging metabolite protons. Through chemical exchange, this saturation is transferred to water protons leading to a reduced water signal, which can be measured by a standard MRI sequence. A CEST experiment entails acquiring multiple images with preparations of different saturation frequencies (fig. S1, C and D), allowing the construction of the so-called Z-spectrum or CEST spectrum by plotting the (normalized) saturation signal as a function of the saturation frequency for each image pixel (Fig. 1C). The Z-spectrum exhibits a peak at 0 parts per million (ppm) (=water frequency) due to direct water saturation. To eliminate the effect of direct water saturation, an asymmetry spectrum MTR_asy_ can be calculated. The MTR_asy_ spectrum of the liquid endosperm of the pea seed shows two peaks at approximately 1 and 3 ppm. The peak at 1 ppm corresponds to the exchanging protons of the hydroxyl group (*27*). As most of these are found in sugar molecules, it is referred to as the “sugar signal.” The 3-ppm peak is attributed to the exchanging amine protons and interpreted as the amino acid signal (*28*). We calculated a sugar CEST signal map by integrating the corresponding peak of the MTR_asy_ spectrum in the range from 0.6 to 1.8 ppm for each pixel and, in the same way, an amino acid CEST map by integrating the peak in the range from 2.2 to 3.4 ppm. Further details of the CEST experiment are given in texts S1 and S2. The maps revealed homogeneous distributions of both sugars and amino acids inside the endosperm (Fig. 1C). When applied to the same seed under the same conditions, the classical CSI experiment delivered spectra showing only distinguishable peaks of two amino acids and overlapping resonances of sugars (Fig. 1D), due to a decreased in vivo CSI substance sensitivity caused by magnetic field inhomogeneities within the sample. In an NMR (CSI) spectrum, the resonance frequency of water protons is around 4.7 ppm, while in a Z-spectrum, it is localized at 0 ppm (by definition). Note also that in the NMR spectrum, primarily resonances of nonexchanging protons are observed (for sugars and amino acids, these are often protons bound to carbon atoms in the molecule). Many of these peaks (for sugars and amino acids) are located upfield, i.e., to the right of the spectrum. In contrast, the exchanging protons of the hydroxyl and amino groups, which are detected by CEST, are located downfield (to the left) of the water resonance.

In CSI, the measured metabolite signals are quite small due to their low concentrations compared to water: In the exemplary spectrum in Fig. 1D, the amplitude of the main sucrose peak (normalized by the water signal from a CSI measurement without water suppression) was determined to be approximately 0.13%. In contrast to the direct spectroscopic measurement, the CEST peak in the asymmetry spectrum of the hydroxyl protons reached approximately 30%. The reason for this is that by multiple exchanges of the metabolite protons with the water protons and repeated saturation of the metabolite protons, an accumulated saturation of the water protons is achieved, which is substantially greater than the metabolite concentration itself. Therefore, we obtain a CEST sugar signal that is up to 200 times higher than the sucrose peak (in CSI). This accumulation effect is a crucial feature of a CEST experiment; the higher signal enables faster acquisitions (less averaging) and/or higher spatial resolutions per measurement time compared to direct measurements of low-concentration metabolite protons: The image resolution in our CEST experiments was 50 μm, while the resolution for CSI was much lower (300 μm), despite a longer (240 versus 90 min) measurement time for CSI. The rational for the different resolutions is that CEST measures the significantly larger water signal compared to the low-concentration metabolite signals detected by CSI. To obtain a sufficiently high signal-to-noise ratio (SNR) for CSI, either the measurement time or the voxel size can be increased according to the “SNR scaling law” (it states that the SNR is proportional to the density of the measured protons, the voxel volume, and the square root of acquisition time). Since the measurement time is always limited for in vivo measurements (especially when tracing dynamic changes in metabolism), only lower resolutions (compared to CEST) are usually possible for CSI (see further details in text S3).

During the mid-stage of pea seed development, when the embryo grows but the seed still contains liquid endosperm, the magnetic heterogeneity of the tissues increases, and the application of CSI becomes challenging (*10*). The CSI image of our sample showed significant signal disruption/aberration and no longer accurately reflected the actual spatial distribution of the metabolites (fig. S3B). The reasons for this are given in detail in text S4. In a comparative approach on the same seed, CEST was less affected by magnetic field differences and thus provided more reliable metabolic images (fig. S4D).

As development progresses (maturation), embryo cotyledons are filled with starch and protein and enter the desiccation phase. This is accompanied by a general loss in NMR signal intensity and shorter transverse relaxation times *T*_2_ (*29*). Both CEST and CSI were applicable in imaging the embryo and yielded similar results regarding the distribution of metabolites (Fig. 1, G and H, and fig. S7, E and F). However, CEST provided approximately fivefold higher resolution in a shorter measurement time than CSI (70 μm in almost 2 hours versus 330 μm in 3.5 hours; for details, see text S3). On the basis of these findings, we conclude that CEST overcomes the limitations of the classical CSI approach due to its significantly higher signal sensitivity (CEST accumulation effect) and lower susceptibility to magnetic field inhomogeneities.

### Dynamic imaging using CEST

**Fig. 2.**
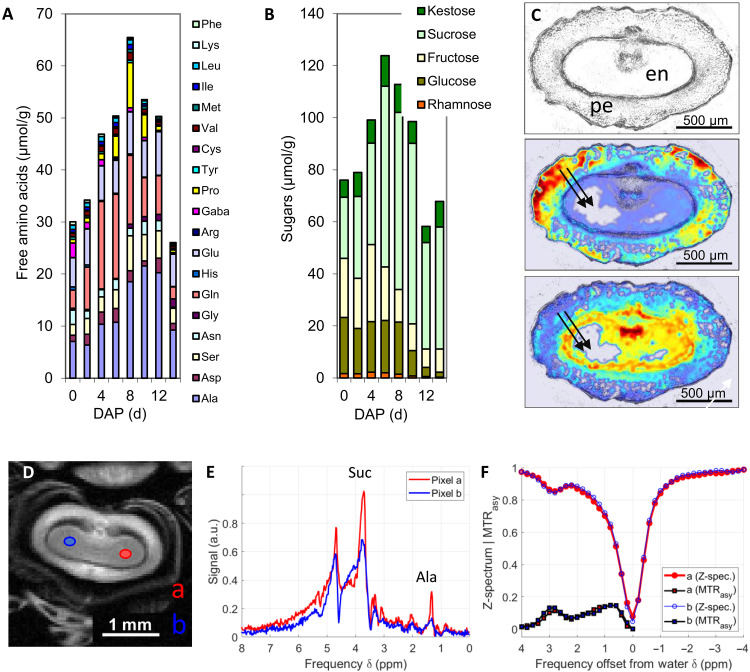
Comparative analysis of metabolites in barley using chromatography, FTIR, CSI, and CEST imaging. (**A** and **B**) Measurements of soluble metabolites in tissue extracts of developing caryopses using chromatography. (**C**) Cross-sectional view of a barley caryopsis at 4 days after pollination (DAP). The endosperm (en) is beginning to cellularize and is mostly liquid at this stage. Distribution of sugars (middle) and amino acids (bottom) in cryosections by Fourier transform infrared (FTIR) imaging and spectral unmixing. Data in relative units with maximum (minimum) values are shown in red (blue). Arrows demonstrate lyophilization artifacts. As solubles recrystallize at crystallization nuclei, some liquid-occupied areas will appear empty. (**D**) NMR imaging of a barley caryopsis. (**E** and **F**) Representative CSI (E) and CEST spectra (F) from the endosperm regions (a) and (b) as shown in (D). pe, pericarp.

Sugars and amino acids are the most abundant metabolic compounds delivered to the largest storage organ of cereals (endosperm), and evaluating their content and tissue-specific distribution is crucial for studying grain quality and yield formation (*30*). In our experiment on barley (*Hordeum vulgare*), conventional methods were used to quantify the composition of grains (Fig. 2, A to C), but destructive sampling interrupts development and prevents monitoring. To our knowledge, there are no available methods to monitor the dynamics of these metabolites in growing cereal grains. To overcome these limitations, we adapted CEST MRI for measurements on grains attached to the intact spike. MRI clearly identified an individual grain and the endosperm within the grain. The endosperm is surrounded by the pericarp and shaped in two symmetrical wings. As expected, the CEST spectra, acquired in these wings, matched very well (mean deviation of the two Z-spectra: 2.6%), whereas the CSI spectra clearly differ from each other (mean deviation over 38%) (Fig. 2, E versus F). Magnetic field inhomogeneities made it impossible to obtain reasonable metabolite maps using CSI, as explained in text S4.

**Fig. 3.**
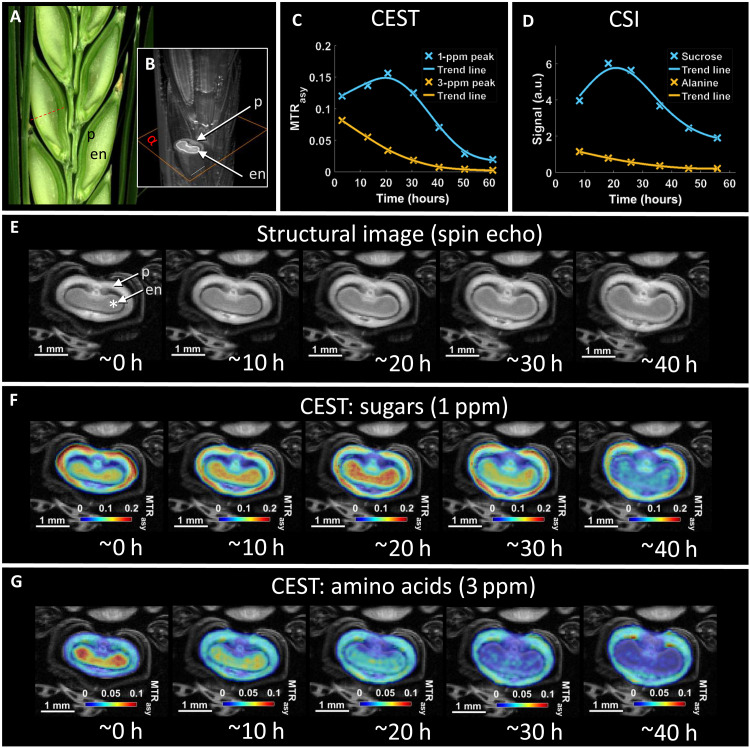
Dynamic imaging of growth, sugar and amino acid distribution within the barley grain. (**A**) Photographic image of the developing barley spike, pictured by light microscopy. (**B**) Noninvasive assessment of developing caryopses using MRI. Scale bar, 1.5 mm. (**C** and **D**) Dynamic course of the CEST-MTR_asy_ and CSI signal for sugars and amino acids in the voxel (*) labeled in (E). (**E**) Structural MRIs showing the growth of the caryopsis. (**F** and **G**) Imaging of the alternating sugar (F) and amino acid distribution (G) during the growth of the caryopsis using CEST. Please also see movie S1. p, pericarp.

We next measured the growth [using three-dimensional (3D) MRI] and metabolite distribution across the caryopsis over time. For this, the sugar and amino acid signal progression in endosperm was monitored in parallel by both CEST and chemical shift measurements during 60 hours (Fig. 3, A to D). Only CEST enabled reliable imaging (Fig. 3, F to G). During the experiment, the endosperm size grew continuously (Fig. 3E). At the beginning of the monitoring, CEST revealed high levels of sugars in both the endosperm and pericarp of the grain, and amino acids were preferentially localized in the endosperm (Fig. 3, F and G). As the caryopses grew, the accumulation of sugars in the endosperm peaked at approximately 20 hours after the start of the experiment, while the level of amino acids in the endosperm continuously decreased. The metabolically active endosperm became increasingly deficient in amino acids during development. The pericarp maintained a relatively constant level of sugars during the observation time (Fig. 3F). The different alteration of sugars and amino acids in the endosperm versus pericarp is consistent with previous findings, which have characterized the endosperm as metabolically active, proliferating tissue, whereas the pericarp undergoes cessation of cell division and progression of programmed cell death (*20*, *30*). Last, a time-lapse movie was recorded, which displays the never-before-seen dynamics of sugars and amino acids in a growing grain (movie S1). Notably, the CEST maps (Fig. 3, F to G) acquired at start of the experiment (0 hours) are in good agreement with the distributions quantified by Fourier transform infrared (FTIR) microscopy, which was performed in cryosections of caryopses at the grain filling stage (Fig. 2C).

### Elucidating the consequences of genetic engineering using CEST

We hypothesized that CEST could successfully be applied to detect variations in assimilate supply and distribution in mutants and transgenics, thereby elucidating the consequences of genetic interventions in seed development. *HvSWEET11b* (*Sugars Will Eventually be Exported Transporter 11b*) has been shown to function as a dual transporter operating in the export of sugars and cytokinins from the maternal grain parts toward the endosperm (*16*). Homozygous *sw11b-31* mutant plants produced fertile florets but grainless (empty) spikes. Here, we applied CEST, revealing early aberration in the *HvSWEET11b*-mutant grains (fig. S8). The mutant endosperm was largely underdeveloped and unable to occupy the space provided by expanding maternal tissues. Sugars accumulated in the nucellar projection and ventral regions of the pericarp of the *sw11b-31* grains but less so in the endosperm (fig. S8G). Such an aberrant “redistribution” of sugars clearly reflects disturbed allocation of sugars, eventually causing grain abortion.

### Broad applicability of CEST to study economically important crops

**Fig. 4.**
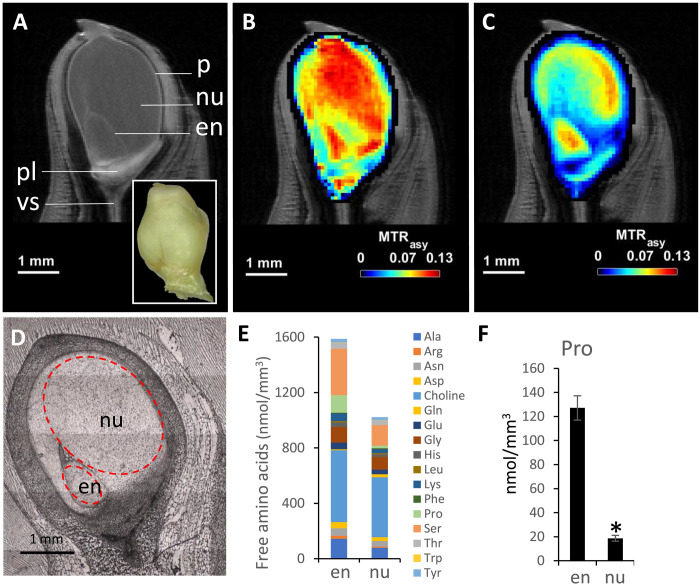
Noninvasive imaging of sugar and amino acid distribution by CEST and metabolite quantification by LC-MS in the early maize kernel. (**A**) MRI reference image visualizing the internal structure of the maize kernel (6 DAP). Photographic image of intact kernel on insert. (**B** and **C**) CEST image of sugars (B) and amino acids (C) in the living maize kernel. Image resolution, 40 μm. (**D**) Cryosection of the kernel used for microdissection of the tissues for detection of free amino acids; red dashed line indicates dissected regions. (**E**) Concentration of free amino acids measured in microdissected endosperm and nucellus using LC-MS. (**F**) Comparison of proline (Pro) level measured in microdissected endosperm and nucellus; *t* test, *P < 0.05; *n* = 3. cp, central placenta; ls, locule and seeds; nu, nucellus; pl, placental-hilar-funicular region.

Next, we tested the applicability of CEST to study a model for which in vivo metabolic features have thus far been inaccessible: the early developing maize kernel (*31*, *32*). At an early stage, the filial part of kernel (endosperm) expands, while the maternal part (nucellus) degenerates (*33*). The interplay between nucellar elimination and endosperm development is relevant for seed size control (*34*). In the present study, CEST visualized a distribution of both sugars and amino acids within the living kernel [6 days after pollination (DAP)] (Fig. 4, A to C). Moreover, we found gradients in the distribution of amino acids with maximum values in the endosperm and peripheral regions of the nucellus (Fig. 4C). Signal intensity differences of approximately 50% indicated a higher abundance of amino acids in the endosperm than in the nucellus. This finding was confirmed by manually dissecting the tissues and conducting a liquid chromatography–mass spectrometry (LC-MS)–based analysis (Fig. 4, D to F). The strongest (approximately sevenfold) enrichment in the endosperm was observed for proline (multifunctional amino acid that can play a significant role in plant development and stress responses) (*35*).

**Fig. 5.**
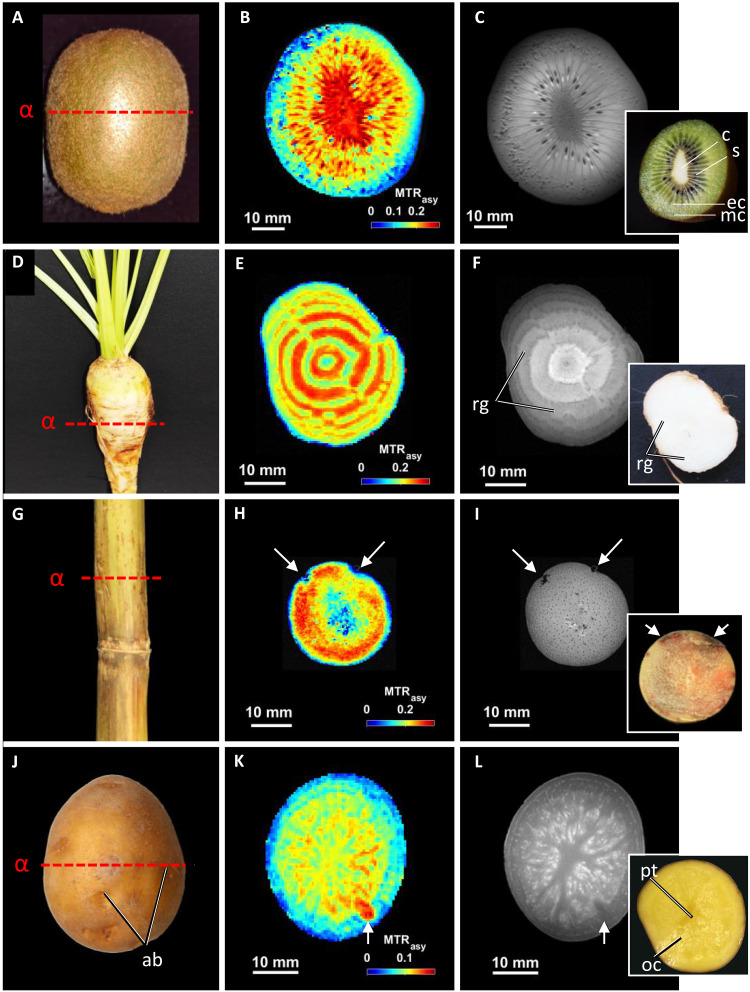
Noninvasive imaging of sugar distribution in plant sink organs. (**A**) Kiwi fruit. Dashed line (α) indicates the orientation of the slice measured by MR. (**B**) CEST image of sugars. (**C**) MR reference image and cross section on insert (photographic image). (**D**) Sugar beet. Dashed line (α) indicates the orientation of the slice measured by MR. (**E**) CEST image of sugars. (**F**) MR reference image and cross section on insert (photographic image). (**G**) Stem of sugarcane. The dashed line (α) indicates the orientation of the slice measured by MR. (**H**) CEST image of sugars. (**I**) MR reference image and cross section on insert (photographic image). Arrows indicate tissue damage. (**J**) Potato tuber (cultivar Nova). The dashed line (α) indicates the orientation of the slice measured by MR. (**K**) CEST image of sugars. Arrow indicates axillary bud. (**L**) MR reference image and cross section on insert (photographic image). Resolution of all CEST images, 500 μm. ab, axillary buds; c, core; ec, endocarp; mc, mesocarp; oc, outer core; pt, pith; rg, rings; s, seeds.

To explore the applicability of CEST to much larger samples, we examined several types of plant sink organs including fruits (kiwi: *Actinidia deliciosa*), taproots (sugar beet: *Beta vulgaris*), stem (sugar cane: *Saccharum officinarum*), and tubers (potato: *Solanum tuberosum*) (Fig. 5). These crops are of economic importance worldwide and have previously been subjected to structural MRI (*36*–*40*). Kiwi fruits are relevant model for plant and medical research and examined by various conventional metabolite analyses (*41*, *42*). CEST images of kiwi fruits unveiled previously unknown metabolic arrangements within the true berry (Fig. 5B and fig. S9). Both sugars and amino acids accumulate within the fruit core; another ring-like accumulation occurs in the endocarp (fig. S9).

An anomalous type of secondary root thickening growth (taproot) is found in all members of the genus *Beta*, including sugar beet (*B. vulgaris* spp. *vulgaris*) with most agronomical relevance (*43*–*45*). Using CEST, we uncovered here the in vivo pattern of sugar distribution (Fig. 5E). Sugar deposition shows a ring-like, periodic pattern, reflecting the alternating zones of vascular and parenchymatic tissues. This pattern corresponds well with biochemical, molecular, infrared, and MRI studies on developing taproot (*5*, *45*, *46*).

Pattern of sugar accumulation in sugarcane stem (culm) can also be visualized using CEST (Fig. 5H) (validated by CSI in fig. S10). Sugarcane showed a completely different pattern compared to sugar beet. CEST offers to trace the metabolic and developmental state of the culm internodes, which are known to affect sugar yield/content (*47*–*49*). Further, the CEST approach could be useful for investigating sink-source relationships at the tissue level. In our experiment with potato tubers, CEST revealed clear gradient distributions of sugars along with structural elements (pith, outer core, and cortex) (Fig. 5K). Some local maximum in sugar accumulation colocalized with the axillary buds (arrows in Fig. 5, K and L). Moreover, CEST clearly differentiated the metabolite compositions of various other potato cultivars (fig. S11).

## DISCUSSION

Our studies demonstrate that CEST is a powerful MRI approach that facilitates in vivo metabolic analysis in plants, allowing microscopic resolution and dynamic assessment of sugar and amino acid distribution despite the magnetic heterogeneity of the samples. Its application to various crops demonstrates that CEST is a species-, variety-, and organ-agnostic approach to noninvasively visualizing metabolites without the need for prior labeling or sample processing. While several phenotyping methods for plants are in use (*5*, *49*–*51*), few are capable of spatiotemporal assessment of metabolites in crops. We showed metabolite dynamics in growing seeds, which is impossible using conventional techniques. Knowledge of the spatiotemporal dynamics of sugars/amino acids in sink organs is highly sought after by breeders. Their distribution influences mass transport and metabolism in many ways; this knowledge ultimately flows into crop improvement.

The CEST offers unprecedented opportunities for monitoring dynamic changes in metabolites in living plants. It is particularly important for a deeper understanding of trait formation and supporting breeding research by in vivo testing of metabolic responses to genetic engineering and/or developmental alterations. Visualization of metabolite dynamics in living plants is a desired tool to bridge structural and metabolic interactions in plant responses to ever-changing environments. Thus, the introduction of CEST, which visualizes internal tissue structure and metabolite dynamics while avoiding tracers using only one technological platform, MRI, is an important milestone toward this goal.

To explain how and why our CEST approach overcomes the methodological limitations of classical CSI, we compare these two NMR methods when applied to the same samples from different organs of different plants. Magnetic field inhomogeneities usually occur in plants (*14*) and can significantly reduce the spectral sensitivity of conventional in vivo CSI. They can also cause variations in *T*_2_^*^ (observed *T*_2_ relaxation including the effect of magnetic field inhomogeneities within the sample) across the whole plant sample, resulting in distorted and unreliable metabolite maps. In contrast, CEST is much less sensitive to *T*_2_^*^ variations, which is a major advantage (see text S4). In addition, CEST eliminates the need for water suppression, which is crucial for obtaining informative CSI spectra, as the dominant water signal otherwise overlaps with the metabolite resonances and interferes with their analysis. However, sufficient water saturation is only achievable without magnetic field shifts over the sample, which is often challenging in plants. This problem is circumvented in CEST because magnetic field inhomogeneities induce frequency shifts of the Z-spectra over the sample, but they can (and have to) be corrected in postprocessing (*B*_0_ correction). This *B*_0_ correction requires the acquisition of a *B*_0_ map: For that, we always measured a second CEST spectrum with short saturation time and small saturation power [so-called water saturation shift referencing (WASSR) spectrum]. This spectrum shows no CEST effect but only direct water saturation and can therefore be used to determine the water frequency shift. This is used to correct/shift the Z-spectra before the asymmetry spectrum is calculated. A more detailed explanation is described in texts S1 and S4. This feature allows the acquisition of CEST metabolite maps in samples with large magnetic field shifts, greatly expanding the range of CEST applicability compared to CSI.

Furthermore, CEST enables the acquisition of highly spatially resolved metabolite maps, whereas CSI is typically performed with low spatial resolutions (large voxels) to avoid long measurement times. Moreover, the low metabolite signals in CSI (approximately three orders of magnitude less than the signal of water measured by CEST) demand many image averages to reach a sufficient SNR, resulting in extended measurement times. These drawbacks are not present in CEST because it is an imaging technique that measures the higher (saturated) water signal.

Currently, CEST does not provide sufficient chemical resolution to accurately differentiate among individual sugars or amino acids, which is theoretically possible with CSI. However, the conversion of free amino acids and sugars into proteins and starch could be observed in the endosperm of the growing barley caryopsis via CEST by detecting a decreasing CEST signal. The reason for this must be that the protons of the hydroxyl and amino groups still exist but, if they are bound in the starch or protein molecule, no longer exchange with free water protons and therefore cannot contribute to the CEST signal.

For future developments, more quantitative CEST experiments such as quantification of exchange rate using varying saturation power (QUESP) (*52*), which enable the determination of concentrations, or the application of exogenous CEST contrast agents such as iopamidol is promising (*53*). In addition, the extension from 2D to 3D CEST presents an interesting prospect for metabolite mapping in 3D.

## MATERIALS AND METHODS

### Plant material

Wild-type barley (*H. vulgare* L., cultivar Barke, Golden Promise, and Bowman) plants and SWEET11b-mutant plants (*16*) were grown under standard greenhouse conditions at 18°C with 16 hours of light and a relative air humidity of 60%. Determination of developmental stages for developing seeds and tissue isolations were performed as described in (*54*). Wild-type pea (*P. sativum*, cultivar Erbi) plants were grown under a 16-hour light/19°C 8-hour dark/16°C regime. Determination of developmental stages for developing seeds and tissue isolation were performed as described in (*10*). Maize (*Zea mays*, cultivar B73) plants were cultivated in a greenhouse under natural light supplemented with lamps to provide a 16/8-hour photoperiod and an approximate light intensity of 800 μmol photons m^−2^ s^−1^. Temperature was controlled at 23°C (day) and 18°C (night). Plants were hand-pollinated for determination of developmental stages (DAP). Sugarcane (*S. officinarum*) and kiwi (*A. deliciosa*) fruits were at their commercial maturity bought from local grocery store. Potato (*S. tuberosum*) tubers and sugar beets (*B. vulgaris*) were taken from field trail of Leibniz Institute of Plant Genetics and Crop Plant Research (IPK) Gene Bank performed in 2022.

### Biochemical analysis of maize kernels

Kernel samples (6 DAP) were frozen in liquid nitrogen, embedded in Tissue-Tek cryomolds, and cryosectioned (12 μm) with a cryotome and using adhesive foils as in (*55*). After lyophilization of sections, the regions comprising either nucellus or endosperm tissues were manually dissected under a stereomicroscope and transferred to 0.5-ml tubes. For background correction, a minor part of adhesive foils (not covered with any tissue/material) was also separated. Following extraction with methanol-chloroform-water mixtures, the extract was used for the detection of free amino acids using ultra performance LC (Vanquish focused) coupled to QExactivePlus hybrid quadrupole-orbitrap mass spectrometer [full procedure described in (*56*)]. An external calibration scheme was applied using authenticated standards (Merck, AAS18-5ML) for quantification. All quantified data were related to the dissected areas (in cubic millimeters).

### Biochemical analysis of barley and pea seeds

Extraction and analysis of soluble sugars and free amino acids were performed as detailed earlier (*57*). Briefly, samples were powdered in liquid N_2_ and extracted with ethanol. Free amino acids were derivatized and measured using high-performance LC, and soluble sugars were separated by ion chromatography coupled to amperometric detection. Identification and quantification were based on authenticated standards.

### FTIR microspectroscopy

Samples were frozen in liquid nitrogen and embedded in Tissue-Tek cryomolds using Tissue-Tek O.C.T. (Sakura Finetek) at −20°C. Embedded tissue were cross-sectioned (16 μm) with a cryotome CryoStar NX7 (Thermo Fisher Scientific) and transferred onto MMI membrane ribonuclease-free slides (Molecular Machines & Industries). Tissue sections were lyophilized and stored in darkness at room temperature until analysis. These slides also were used for internal standardization as described below.

Imaging was performed using a Hyperion 3000 FTIR microscope (Bruker Optics) coupled to a Tensor 27 FTIR spectrometer (Bruker Optics) with an internal mid-infrared source. The focal plane array detector (64 pixels by 64 pixels) was used in transmission mode. The imaging system was continuously purged with dry air. FTIR images were recorded in the spectral range of 3900 to 800 cm^−1^ at a spatial resolution of 11 μm and a spectral resolution of 6 cm^−1^ using 3.5× (15× for high-detail images; 5.5-μm digital resolution with 2-pixel by 2-pixel binning) infrared magnification objectives (Bruker Optics). Each spectrum comprised 64 coadded scans. A reference of a single focal plane array window of the empty light path was acquired before image acquisition and automatically subtracted from the recorded image by the software OPUS (Bruker Optics). Atmospheric absorptions of water vapor and CO_2_ were corrected by OPUS during image acquisition. OPUS files were imported into MATLAB (The MathWorks Inc., Natick, MA, USA) as ENVI files using the multiband-read function or by the irootlab toolbox (*58*). Spectral features such as amino acids and soluble sugar fingerprints along with baseline features were extracted using an extended multiplicative signal correction model to unmix the spectral fingerprint as described earlier (*55*). The spectral signal defined as amino acids was modeled using pure compound spectra of alanine, cysteine, glutamine, and phenylalanine whereas soluble sugar signals are derived from sucrose, glucose, and raffinose spectra. Additional reference spectra for other compound groups were modeled alongside these to recreate the measured image spectra. The image signals for both groups represent the absorbance intensity sum of these individual representative reference spectra that were used for modeling.

### NMR hardware and software

Imaging experiments on barley, pea, and maize were performed on an Avance III HD 400 MHz NMR spectrometer (Bruker BioSpin), using cryogenically cooled saddle coils with inner diameters of 5 and 10 mm (Bruker BioSpin). For specimens with larger size (kiwi, sugar beet, and potato), measurements were performed on an Avance Neo 500 MHz Super Wide Bore NMR spectrometer (Bruker BioSpin) with a birdcage ^1^H quadrature probe head with an inner diameter of 66 mm (Bruker BioSpin).

### CEST measurements

For the CEST experiments, a 2D rapid acquisition with relaxation enhancement (RARE) sequence prepared for saturation transfer was used. The preparation consisted of one single radio frequency pulse with constant amplitude (block pulse) for saturation. The pulses were applied with frequencies in steps of 0.2 ppm, which equals 80 Hz (100 Hz) for the 400-MHz (500 MHz) scanner, over a range of −5 to +5 ppm leading to 51 images. This range is sufficient for detecting the two peaks around 1 and 3 ppm of the hydroxyl and amino group for sugars and amino acids. A saturation power of 2 μT was used; except in the measurement of the kiwi, sugar beet/cane, and potatoes, a power of 2, 3, and 2.5 μT, respectively, was applied. Higher saturation powers were avoided to prevent broad water peaks and strong magnetization transfer contrast. Saturation pulse lengths between 150 and 500 ms were used, depending on the experiment. Experimental optimization revealed that these short saturation times were already sufficient to achieve adequate CEST contrast (see fig. S2). In addition, these short times minimize the undesired *T*_1_ and magnetization transfer contrast effect on the CEST contrast. The reference image for normalization was obtained with an off-resonant saturation at −100 ppm. For the WASSR method (*59*), further 51 images were acquired with low saturation powers at offsets ranging from −1 to +1 ppm with steps of 0.05 ppm. In most experiments, the WASSR preparation consisted of a single 100-ms 0.4-μT block pulse. The slice geometry (orientation, slice thickness, field of view, and resolution) was adjusted to the examined sample. The slice thicknesses were in a range of 400 μm (young pea) and 2000 μm (kiwi). The measurements of the smaller seeds and grains on the 400-MHz scanner had a spatial resolution of 50 to 80 μm, the kiwi/sugar beet/sugar cane/potatoes were measured with a resolution of 500 μm. The resolution of the CEST images of mid-stage pea seeds, barley, and maize grains was increased in postprocessing using a high-resolution reference image of 40 μm (for keyhole technique, see text S5) (*60*). The repetition time of every experiment was 1 s (except for sugar beet/potatoes, 750 ms), and the echo time was always chosen as short as possible (2.6 to 5.1 ms) restricted by the pulse and gradients lengths. For acceleration of the image acquisition, RARE factors of 4 or 8 were used. In addition, one structural reference image with same sequence parameters but RARE factor of 1 was always acquired for visualization of the examined slice. The total measurement times of all CEST (and corresponding WASSR) experiments are listed in table S1.

The analysis and postprocessing of the CEST images were conducted using custom MATLAB (The MathWorks Inc., Natick, MA, USA) scripts. The Z-spectra were corrected pixel by pixel by shifting them (using a smoothing spline interpolation method) around the *B*_0_ frequencies determined by the WASSR measurement. Subsequently, the asymmetry spectrum MTR_asy_ was calculated using eq. S1 (see text S1). The area of MTR_asy_ over a range of offsets from 0.6 to 1.8 ppm (2.4 to 3.4 ppm) was used to depict the CEST effect for sugars (amino acids) in color-coded maps.

### CSI measurements

CSI measurements were performed with the same slice orientations as the corresponding CEST images. They were carried out by measuring free induction decays (FID mode), with an acquisition of 512 data points over a bandwidth of 10 ppm resulting in an acquisition time of 128 ms. CSI measurements were conducted with (and sometimes additionally without) water saturation. For water saturation, a VAPOR (variable pulse power and optimized relaxation delays) suppression scheme with frequency-selective hermite pulses (bandwidth of 150 to 350 Hz depending on the sample) was used (*61*). A Hamming-weighted acquisition scheme was used to reduce the total scanning time and to improve the point spread function (reduction of sidelobes). The processing of the measured NMR data was performed using an in-house written MATLAB script. The spectra in this work are shown in absolute mode. The color-coded metabolite maps were calculated by simply integrating over the corresponding metabolite peaks in the NMR spectra [over the range from 3.3 to 4.4 ppm (1.3 to 1.7 ppm) for sugar/sucrose (alanine); sophisticated analyses such as peak fitting are challenging in in vivo plant spectra and were not performed in this work].

## Supplementary Materials

### The PDF file includes:

Supplementary Texts S1 to S5 Figs. S1 to S11 Table S1 Legend for movie S1 References
