# Supplementary material for: Metabolic imaging in living plants: A promising field for chemical exchange saturation transfer (CEST) MRI
Source: Sci Adv. 2024 Sep 18;10(38):eadq4424. doi: 10.1126/sciadv.adq4424 (PMC11409970; doi:10.1126/sciadv.adq4424)
Supplement: Supplementary file 1 — Supplementary Texts S1 to S5 Figs. S1 to S11 Table S1 Legend for movie S1 References [file sciadv.adq4424_sm.pdf]

Science Advances
AAAS

# Supplementary Materials for

## Metabolic imaging in living plants: A promising field for chemical exchange saturation transfer (CEST) MRI

Simon Mayer *et al.*

Corresponding author: Peter M. Jakob, peja@physik.uni-wuerzburg.de; Ljudmilla Borisjuk, borisjuk@ipk-gatersleben.de

*Sci. Adv.* **10**, eadq4424 (2024)
DOI: 10.1126/sciadv.adq4424

**The PDF file includes:**

Supplementary Texts S1 to S5
Figs. S1 to S11
Table S1
Legend for movie S1
References

**Other Supplementary Material for this manuscript includes the following:**

Movie S1

**Supplementary Text 1**

Performing a CEST experiment

A CEST experiment includes the acquisition of many MR images, each prepared with a different saturation frequency (see Fig. S1D). In our experiments the preparation comprises one single RF pulse with constant amplitude (block pulse; see Suppl. Text 2 for more details) followed by the signal acquisition. For that, we used a conventional Cartesian fast spin echo technique (see Methods). Plotting the saturation signal (for each pixel) as a function of the saturation frequency gives the so-called Z- or CEST spectrum (Fig. 1D). The frequency is stated as the relative shift $\Delta\omega$ to the water frequency in parts per million [ppm]. The signal $S(\Delta\omega)$ is normalized to a reference signal $S_{ref}$ without or alternatively with far off-resonant saturation. The Z-spectrum exhibits a peak around 0 ppm due to direct water saturation. The direct water saturation can interfere with CEST effects, especially with those of sugars because their exchanging hydroxyl protons have only a small chemical shift to water of around 1 ppm (27). To eliminate the effect of direct saturation, different evaluation procedures have been developed (62-64). The simplest and most frequently utilized method, which we also applied in this work, is the calculation of the asymmetry spectrum $MTR_{asy}$ (65)

$$\mathrm{MTR_{asy}}(\Delta\omega) = \frac{S(-\Delta\omega) - S(\Delta\omega)}{S_{ref}} \qquad \textbf{(S1)}$$

For calculating this metric, the exact water frequency (0 ppm) is required to choose the two corresponding signals on the opposite sites at $\pm\Delta\omega$. This is of crucial importance because small deviations can already lead to large errors in the asymmetry spectrum, especially for signal close to the water frequency like for sugars at around 1ppm (see Fig. S4). An established method to determine the water frequency ($B_0$ map) is the so-called WASSR approach (59). A WASSR spectrum is basically a Z-spectrum with a short and low saturation. In that case, the CEST effect is negligible and only direct water saturation is measured. Then, the center (= minimum) of the WASSR spectrum corresponds to the desired water frequency.

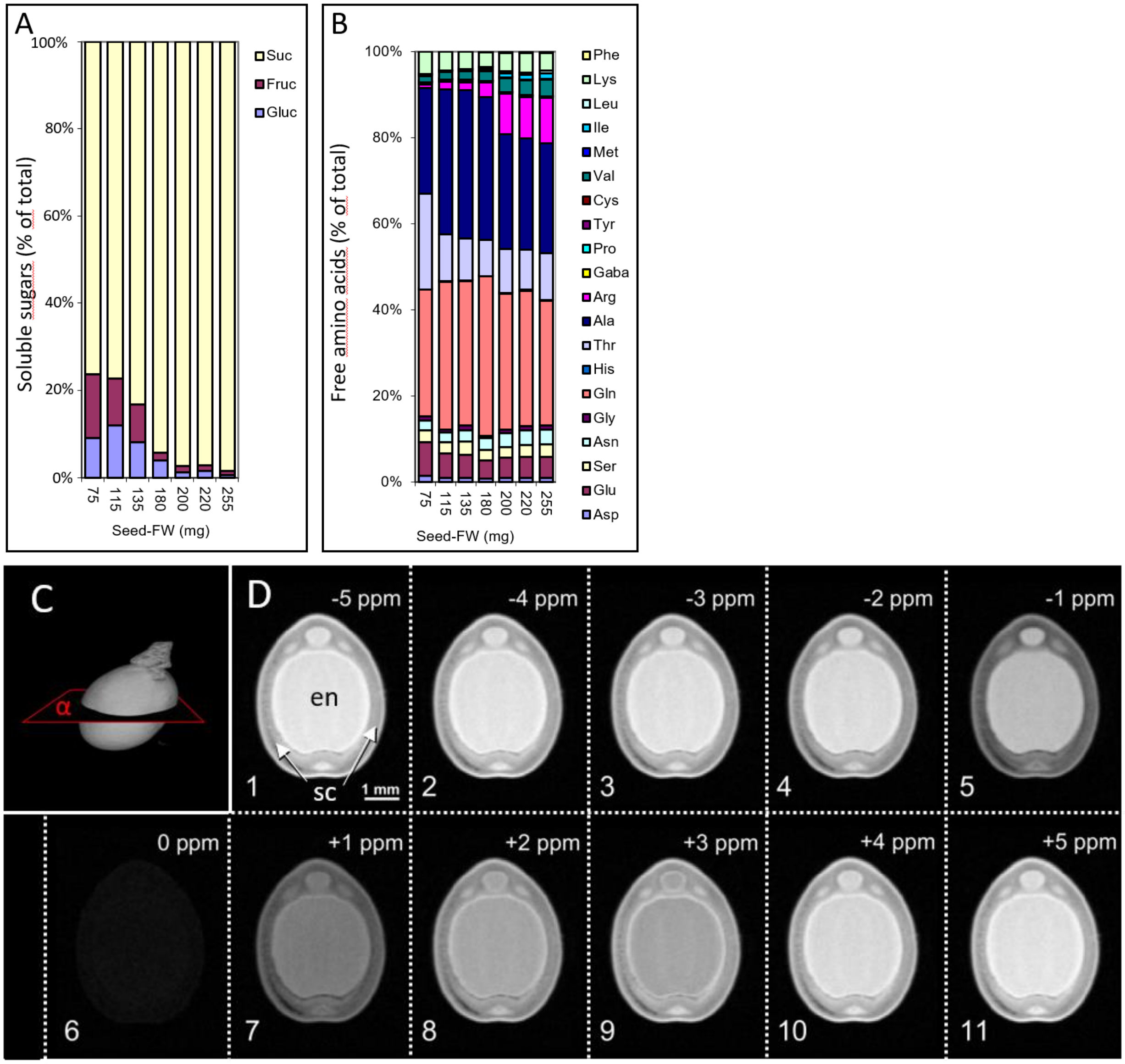


**Supplementary Figure S1: Analysis of liquid endosperm in developing pea seeds. (A, B)** Relative levels of soluble sugars and free amino acids in dissected endosperm measured at distinct developmental stages by chromatography. **(C, D)** 3D model of a pea seed showing the orientation of the slide measured by MRI and shown in **(D1-11)**. A selection of single CEST images with saturation at different frequencies (slice thickness 400 µm). Bar: 1 mm. Abbreviations: en, endosperm; sc, seed coat.

## Supplementary Text 2

Choice of the CEST saturation parameters

In this work, we used continuous wave (cw) CEST: The saturation of our CEST experiments consisted of one rectangular (block shaped) radiofrequency pulse of duration $t_p$, amplitude $B_1$ and frequency offset $\Delta\omega$. For the acquisition of a full Z-spectrum, the measurement is repeated for different frequency offsets $\Delta\omega$. The CEST effect depends on the two saturation parameters $t_p$ (saturation time) and $B_1$ (saturation power).

Although the saturation parameters are given in the method section, we would like to explain why this choice makes sense. For this purpose, we performed CEST measurements on a young pea using different saturation parameters. The outcome is shown in Fig. S2, where measured Z- and asymmetry $MTR_{asy}$ spectra from the liquid endosperm of the pea are presented for various saturation parameters. Each asymmetry $MTR_{asy}$ spectrum shows two peaks, one at about 1 ppm due to the exchanging hydroxyl protons, interpreted as sugar signal, the other nearly 3 ppm due to the exchanging amino protons, interpreted as amino acid signal. If the saturation power is too low, like 1 µT, there is only a slight CEST effect and only small peaks. If the saturation power is too high, the exchanging peaks and the water peak (direct water excitation) become broader and can no longer be easily separated from each other (Fig. S2A).

The CEST effect rises with increasing saturation time (see Fig. S2B) until it reaches a steady state for a sufficiently long saturation time. This is the case because the accumulation of saturation due to multiple exchange is limited/reduced by $T_1$ relaxation of the (saturated) water protons. The CEST effect is therefore influenced by $T_1$ relaxation of the measured substance/tissue. As the influence of $T_1$ on the CEST effect increases with longer saturation time (due to the fact that an extended saturation time leads to a prolonged time for $T_1$ relaxation), our strategy was to favor shorter saturation times. In this particular case of the young pea, appropriate parameters were $B_1$=2 µT and $t_p$=300 ms.

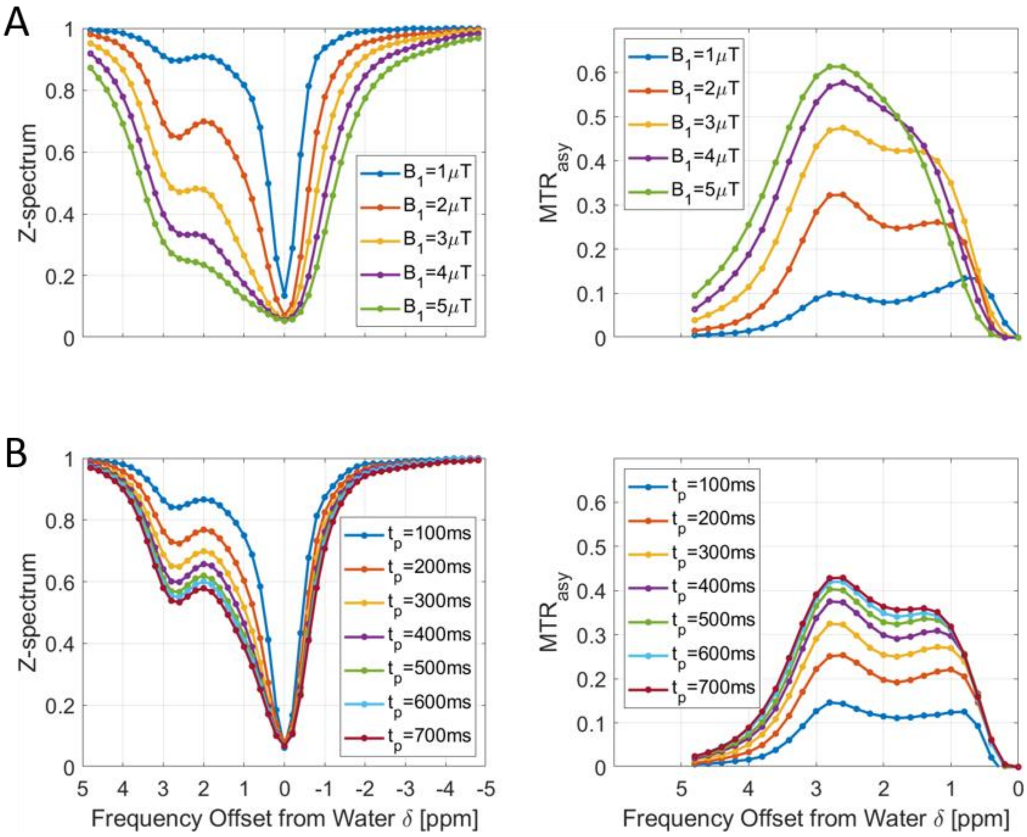


**Supplementary Figure S2: Measured Z-spectra and corresponding asymmetry spectra $MTR_{asy}$ of liquid endosperm of a young pea for different saturation parameters. (A)** Z-spectra for different saturation powers $B_1$ and saturation time $t_p$=300 ms, and the corresponding asymmetry spectra $MTR_{asy}$. **(B)** Z-spectra for different saturation times $t_p$ and saturation power $B_1$=2 μT, and the corresponding asymmetry spectra $MTR_{asy}$.

**Supplementary Text 3**

<u>Spatial resolution of CSI and CEST images</u>

In the following, we explain the reasons for the different spatial resolutions of CSI and CEST images. The resolution of an image is defined as the field of view (FOV) divided by the matrix size of the image.

CSI measurements have long measurement times if high spatial resolved images are acquired. For example, a CSI measurement on a young pea with an isotropic resolution of 100 µm took over 11 hours (10). One reason for this is that a spectrum is acquired for each voxel, which corresponds to an additional measurement dimension in addition to the spatial dimensions. MRI speaking, no (fast) read encoding can be used for image acquisition, but only (slow) phase encoding. On the other hand, CSI directly measures low metabolite signals compared to the water signal usually measured in MRI (as metabolites are less concentrated than water). Therefore, signal averaging is usually required to obtain a sufficient signal to noise ratio (SNR), which can increase the measurement time immensely. For this reason, CSI measurements tend to be performed at lower resolutions compared to MRI, such as our measurement of the young pea with resolution of 300 µm (see Fig. 1). However, lower resolutions correspond to larger voxels, which in turn makes the measurement more susceptible to magnetic field inhomogeneities. In addition, small acquisition matrices lead to an inconvenient point spread function: The point spread function now also exhibits side loops outside the nominal resolution (FOV divided by matrix size), which means that the signal of a voxel is contaminated by signals from neighboring voxels (66).

CEST, on the other hand, is an MRI method. This means that read encoding can be used for image acquisition, which significantly reduces the measurement time. Furthermore, less image averaging is usually required as no small metabolite signals are directly measured, but (saturated) water signal (note the accumulation effect due to multiple exchanges!).

In our work, we have achieved resolutions of up to 50 µm for CEST (see the measurement on the young pea). This is a typical spatial resolution for a MR microscopy measurement, whose resolution is generally in the range of 10 µm to 100 µm (67). The lower resolution limit is usually limited by the signal-to-noise-ratio (SNR), which decreases proportionally with the voxel volume. As the SNR can be increased by longer (repeated) data acquisition, there is always a trade-off between measurement time and resolution. Additionally, microscopy measurements are only possible if optimized NMR devices are available: The measurements in this work were carried out

on high-field devices (400 MHz or 500 MHz); furthermore, a 5 mm cryo sample head for the 400 MHz scanner was available, which is perfectly suited for microscopy measurements.

**Supplementary Text 4**

Influence of magnet field inhomogeneities on CSI and CEST measurements

CEST measurements are significantly less sensitive to magnetic field inhomogeneities compared to CSI. Since these inhomogeneities often occur in plant samples, this represents a decisive advantage of CEST over CSI. In the following, this will be demonstrated and explained using exemplary measurements on a pea at mid-developmental stage (see Fig. S3A). A distinction must be made between magnetic field inhomogeneities across the whole sample and magnetic field inhomogeneities within individual voxels.

1. Magnetic field shifts across the sample

The high water content in living plant tissue results in a strong dominance of water peaks over soluble metabolites in CSI spectra. In classical CSI approach, the water signal must be suppressed for quantitative analysis of metabolites. The most accepted method for water suppression is to use global (over the whole sample) frequency-selective saturation pulses. The success of the experiment depends to a large extent on the efficiency/accuracy of the water suppression.

Fig. S3A shows as an example the reference structural MR image and the corresponding sugar signal distribution measured by CSI of a pea sample (Fig. S3B). Excessive signal can be seen at the edge of the sample (arrowed in Fig. S3B). At these locations, the water signal appears to be insufficiently suppressed and overlaps the signals of the metabolites. The reason for this lies in global magnetic field inhomogeneities (very common in plants), which lead to different shifts of the resonance frequency across the sample. If the local resonance frequencies are strongly shifted (e.g. greater than the bandwidth of the saturation pulses), the water suppression aimed by global frequency-selective saturation pulses is not achieved and the metabolite signals cannot be detected separately.

We measured the field shifts ($B_0$ map) using the WASSR method (59), to demonstrate strong magnet fields inhomogeneities within the sample (Fig. S3C). The frequency shift is shown in Hz. Especially at locations with shifts of the water resonance to lower frequencies (e.g. seen in blue, Fig. S3C), the water signal is insufficiently saturated, leading to erroneous additional signal in the CSI sugar map (Fig. S3B). Such an artifact cannot be corrected by post-processing and represents a significant limitation of CSI for metabolite measurements in plant NMR.

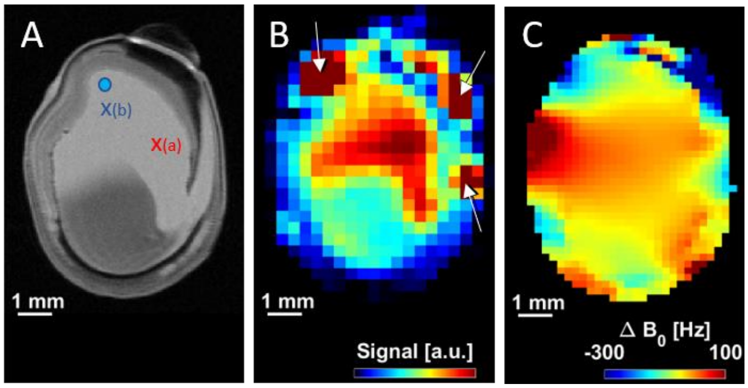


**Supplementary Figure S3: Measurements on a pea at mid-developmental stage for explaining the influence of magnet field shifts on CSI. (A)** Structural reference image of a slice (thickness 400 µm) through a pea. The blue circle indicates the position of the voxel whose Z-spectrum is shown in Fig. S4A,B. The red (a) and blue (b) crosses show the positions of the voxels whose signals are plotted in Fig. S5A,C. **(B)** CSI map of sugar signal. Arrows indicate locations with high signals due to insufficient water suppression. **(C)** $B_0$ map acquired by a WASSR measurement.

CEST spectra could also be affected by magnet field inhomogeneity leading to shifts of the CEST spectra in the frequency/ppm direction (Fig. S4A). This results in erroneous CEST signal maps (see Fig. S4C). However, it can be corrected based on a separately measured $B_0$ map (e.g. using the WASSR method) and shifting the Z-spectra of each voxel based on the respective frequency from the $B_0$ map. This ensures that the water peak of each Z spectrum is actually in the center at 0 ppm, so that a correct asymmetric analysis can now be performed (see Fig. S4B, D). The procedure can be easily performed in post-processing. Frequency shifts within the sample due to magnetic field inhomogeneities can therefore be corrected for CEST measurements.

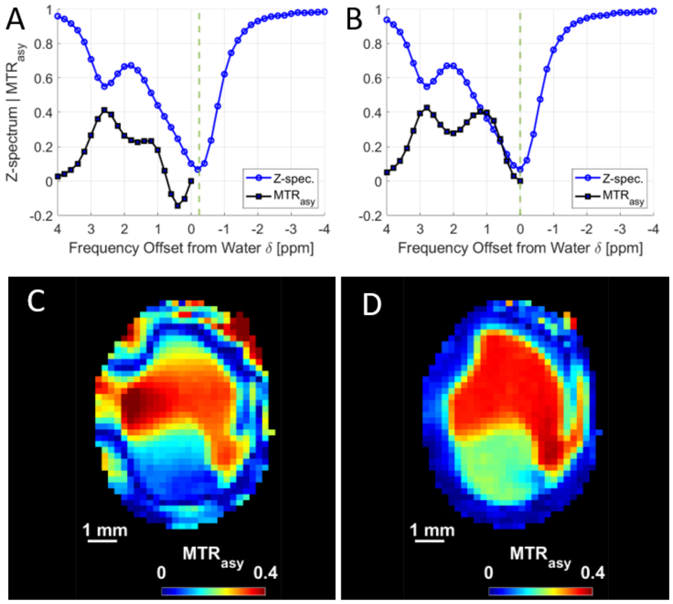


**Supplementary Figure S4: Measurements a pea at mid-developmental stage for explaining the influence of magnet field shifts on CEST. (A/B)** An exemplary CEST spectrum from the endosperm (positions: see blue circle in Fig. S3A) without/with $B_0$ correction. The dashed vertical line shows the position of the water peak. **(C/D)** CEST map of sugar signal without/with $B_0$ correction.

2. Magnetic field inhomogeneities within individual voxels

Another advantage of the CEST approach is to overcome magnetic field inhomogeneities within individual voxels, which is a challenge for CSI. This can be also demonstrated in experiment.

A CSI measurement without water suppression on the same slice and sample was carried out. The signal of two exemplary voxels a and b (positions indicated as crosses in Fig. S3A) from the distinct endosperm region is shown in Fig. S5A. The signal from voxel b decreases faster over time than the signal from voxel a. This is due to a less homogeneous magnetic field in voxel b and leads to a stronger dephasing of the magnetization and a faster signal decay. The signal decrease can be described by an exponential decay with decay time $T_2^*$ (transverse relaxation time). An exponential fit of all signal curves provides a $T_2^*$ map (Fig. S5B). This map illustrates quite well the different magnetic field homogeneities within the individual voxels. These magnetic field inhomogeneities have a direct influence on the CSI spectra (Fig. S5C). As a result, signal gradients

in the CSI metabolite map (Fig. S3B) within the endosperm are detected, but not due to concentration changes of the metabolite, but due to different magnetic field homogeneities.

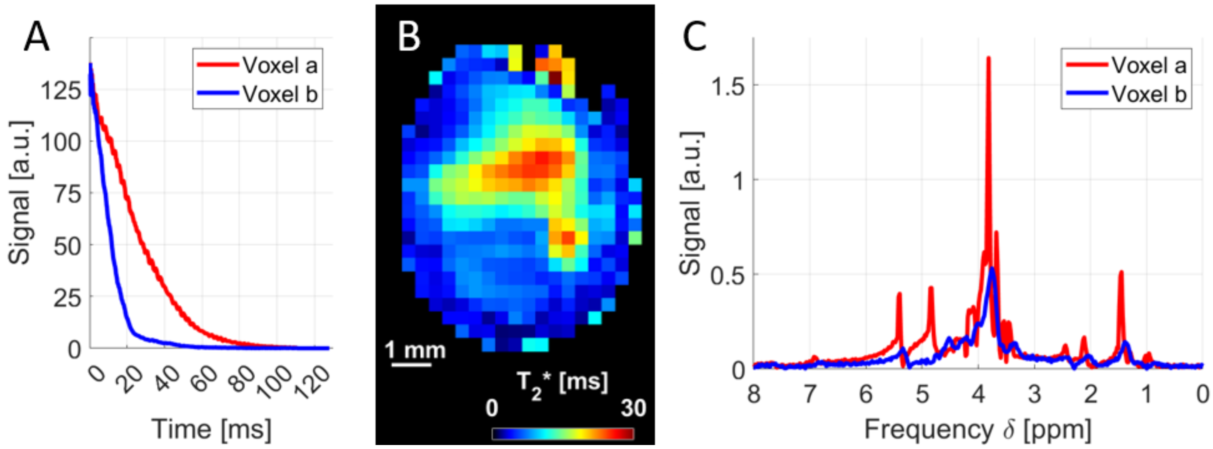


**Supplementary Figure S5: CSI measurement on a pea at mid-developmental stage showing different $T_2$* relaxation within the sample. (A)** Exemplary water signals from two voxels within the endosperm (acquired by CSI without water suppression; position: see red and blue crosses in Fig. S3A) showing different signal decays due to varying magnetic field inhomogeneities. **(B)** $T_2^*$ map obtained by exponential fitting of the water signal. (C) Exemplary CSI spectra (with water suppression) from the two voxels a and b. They differ significantly due to different homogeneities of the magnetic field.

The CEST measurements in this work are less dependent on these different $T_2^*$ relaxations. First, because a spin-echo sequence was used for CEST and this compensates for signal decreases due to static magnetic field inhomogeneities (68). Second, the different signal decays are not detected because CEST imaging requires only a few milliseconds of acquisition time. In contrast, long data sampling times of around 100 ms are necessary for CSI in order to achieve the spectral resolution of the NMR spectra. Third, $T_2^*$ sensitivity is reduced due to smaller voxel sizes and the corresponding lower intra-voxel dephasing compared to CSI.

**Supplementary Text 5**

Spatial interpolation of CSI and CEST images

Due to the typically low spatial resolution of CSI images, the images are often interpolated in post-processing. Since the data are measured in the so-called k-space, the resolution can be formally increased by adding zeros in the k-space periphery (zerofilling, (69)). Subsequent Fourier transformation of the k-space enlarged with zeros generates an image with increased resolution (see Fig. S6A). It should be noted that this does not generate any additional information and is primarily an aesthetic improvement, as an apparently higher resolution is achieved with a reduced partial volume effect.

As discussed in Suppl. Text 3, CEST images typically have a higher spatial resolution than CSI images, but can also be interpolated using zerofilling. In this work, however, a slightly different method was used (if interpolation was applied), the so-called keyhole technique (60): Here, the k-space of each CEST saturation image is not expanded with zeros but with the k-space periphery of a higher-resolved image that was additionally acquired without CEST preparation/saturation. So, the formal resolution of all CEST images is increased and thus, also the CEST metabolite maps calculated from them (see Fig. S6B). Again, no additional CEST information is generated.

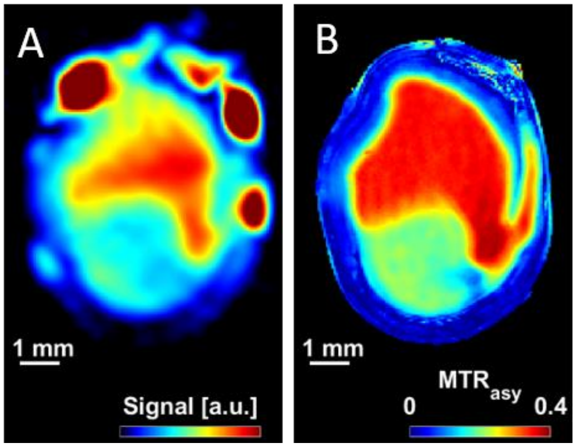


**Supplementary Figure S6: Measurements on a pea at mid-developmental stage for demonstrating spatial interpolation methods. (A)** CSI map of sugar signal with increased spatial resolution by zerofilling in post-processing. **(B)** CEST map of sugar signal with increased spatial resolution by using a keyhole technique in post-processing.

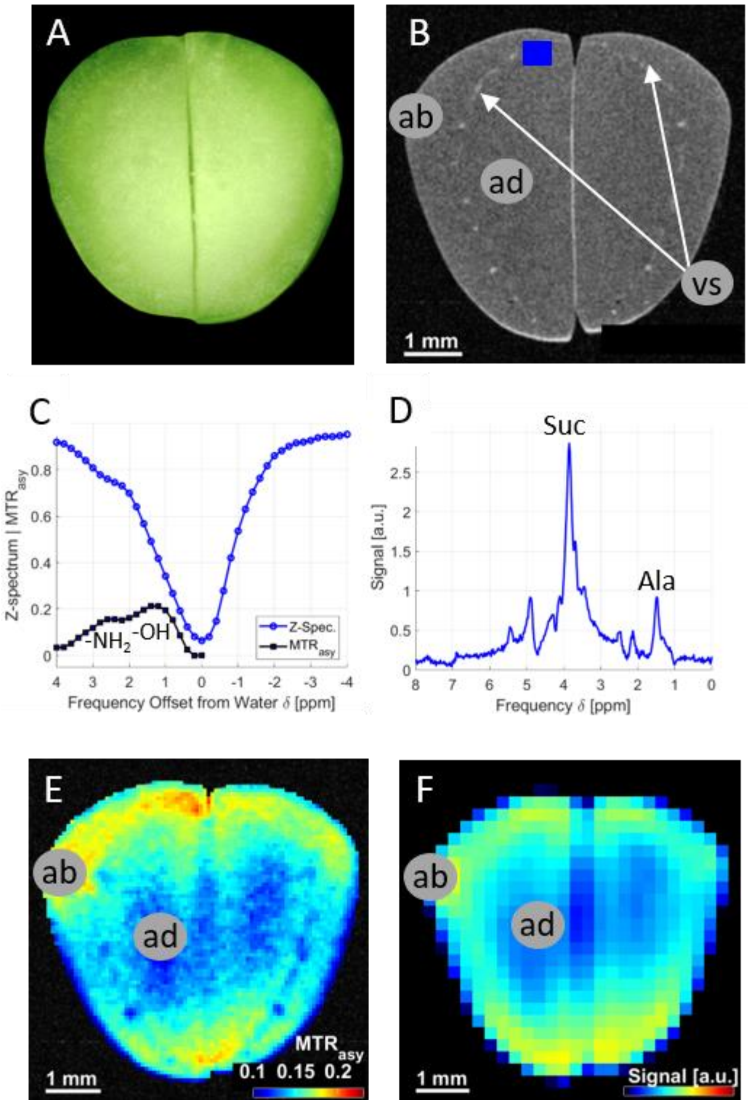


**Supplementary Figure S7. Comparison of CEST and CSI for metabolite imaging in pea embryos at the late developmental stage.**

**(A)** Photographic image showing a slice through an isolated pea cotyledon. **(B)** Tissue structure of the cotyledons as imaged by MRI (slice thickness 800 µm). The blue dot shows the position for exemplary spectral analysis by CEST **(C)** and CSI **(D). (E, F)** Visualization of sugar distribution within cotyledons as measured by CEST **(E)** and CSI **(F)**. Elevated levels of metabolites were detected in the abaxial regions of the embryo. Vascular tissues could not be resolved by CSI. Abbreviations: ab, abaxial parenchyma (faced to the seed coat region of embryo); ad, adaxial parenchyma (faced to the hypocotyl region of embryo); cot, cotyledons; vs., vascular tissues.

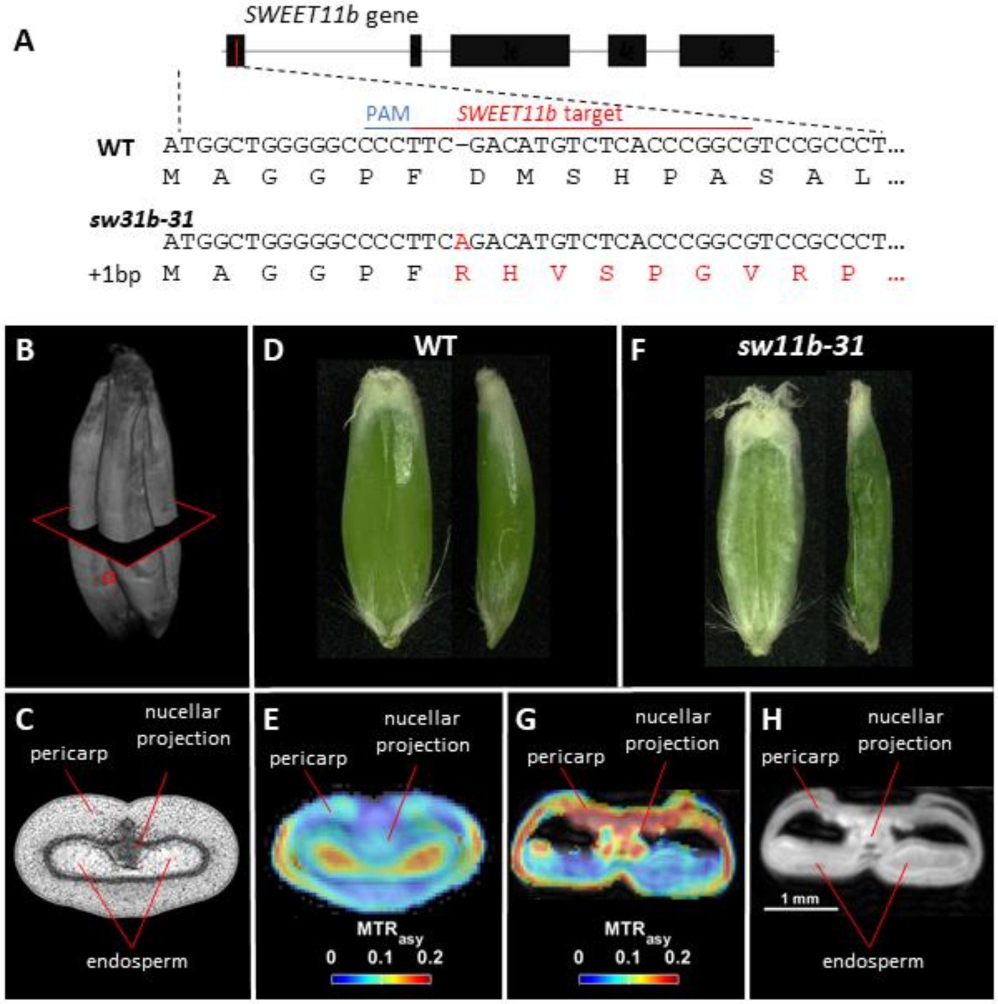


**Supplementary Figure S8. Application of CEST for study of barley mutant *sw11b-31* with deficient grain filling.** (**A**) HvSWEET11b gene model with a position targeted by guide RNA (red box). Exons are shown as black boxes, and introns are shown as lines (above). Nucleotides 1 to 44 and their corresponding protein sequences shown underneath for the wild type (cv. Golden Promise) and *sw11b-31*. Lines over the WT sequence indicate the guide RNA target (red) and protospacer adjacent motif (PAM, blue). A dash in the WT sequence indicates a position for a nucleotide insertion in *sw11b-31*. (**B**) An NMR image of barley grains with a position for virtual sections shown in (**C**) and (**E, G, H**). (**C**) A cross section of a barley grain showing major seed tissues. (**D**, **F**) Grain phenotypes of wild type (**D**) and *sw11b-31* (**F**). (**E, G**) CEST imaging of sugars in wild-type (**E**) and *sw11b-31* grains (**G**). (**H**) Structural reference image through a grain of *sw11b-31*.

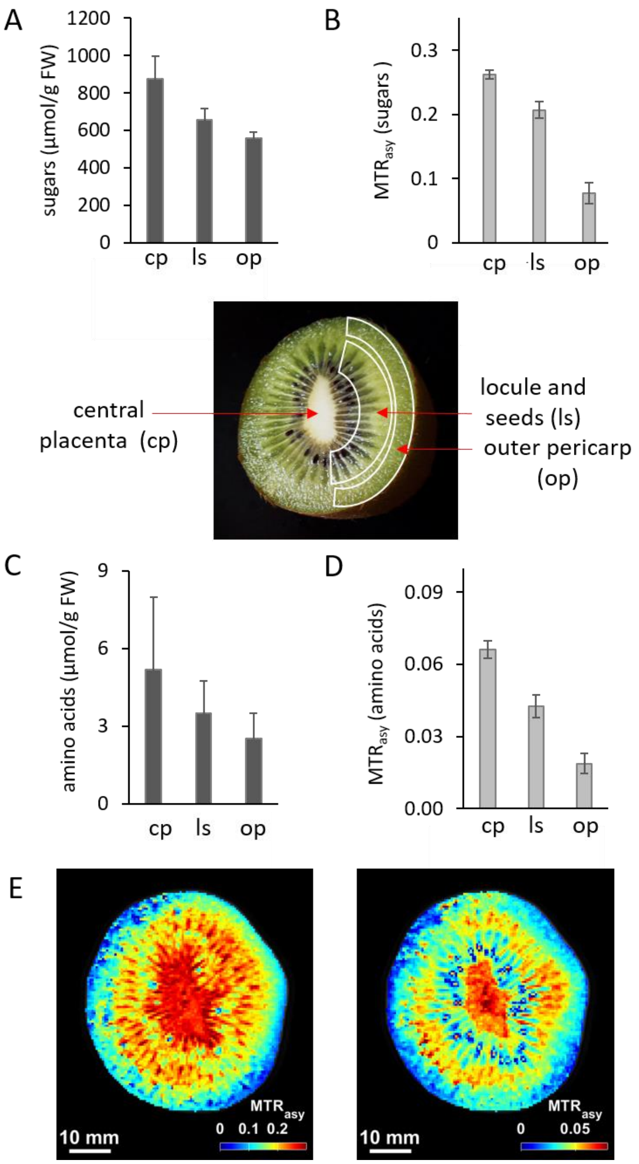


**Supplementary Figure S9. Quantification of sugars and amino acids in kiwi fruit using CEST and chromatography/mass spectrometry.**

(**A,B**) Soluble sugar content in three manually dissected regions of kiwi fruit measured using ion chromatography (**A**) in comparison to values determined by CEST (**B**). **(C,D)** Amino acid content in same regions measured by mass spectrometry (**C**) in comparison to values determined by CEST (**D**). (**E**) Visualization of sugars (left) versus amino acids distribution within cotyledons as measured by CEST. Resolution of all CEST images 500 µm. Data are shown as mean values +/- standard deviation. Number of replicates: n=3 in (**A,C**); n=5 in (**B,D**). Abbreviations: cp, central placenta; ls, locule and seeds; op, outer pericarp.

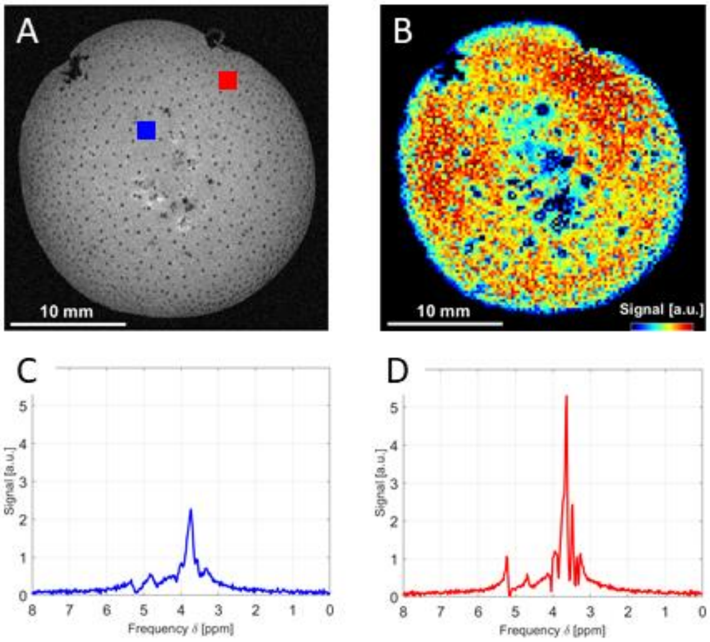


**Supplementary Figure S10. Sugar imaging in the stalk of sugar cane by CSI.**
(**A**) Tissue structure of the sugar cane as imaged by MRI. The blue/red dot shows the position for exemplary spectral analysis by CSI (**C/D**). (**B**) Visualization of sugar distribution within tissues as measured by CSI. (**C/D**) Exemplary CSI spectra.

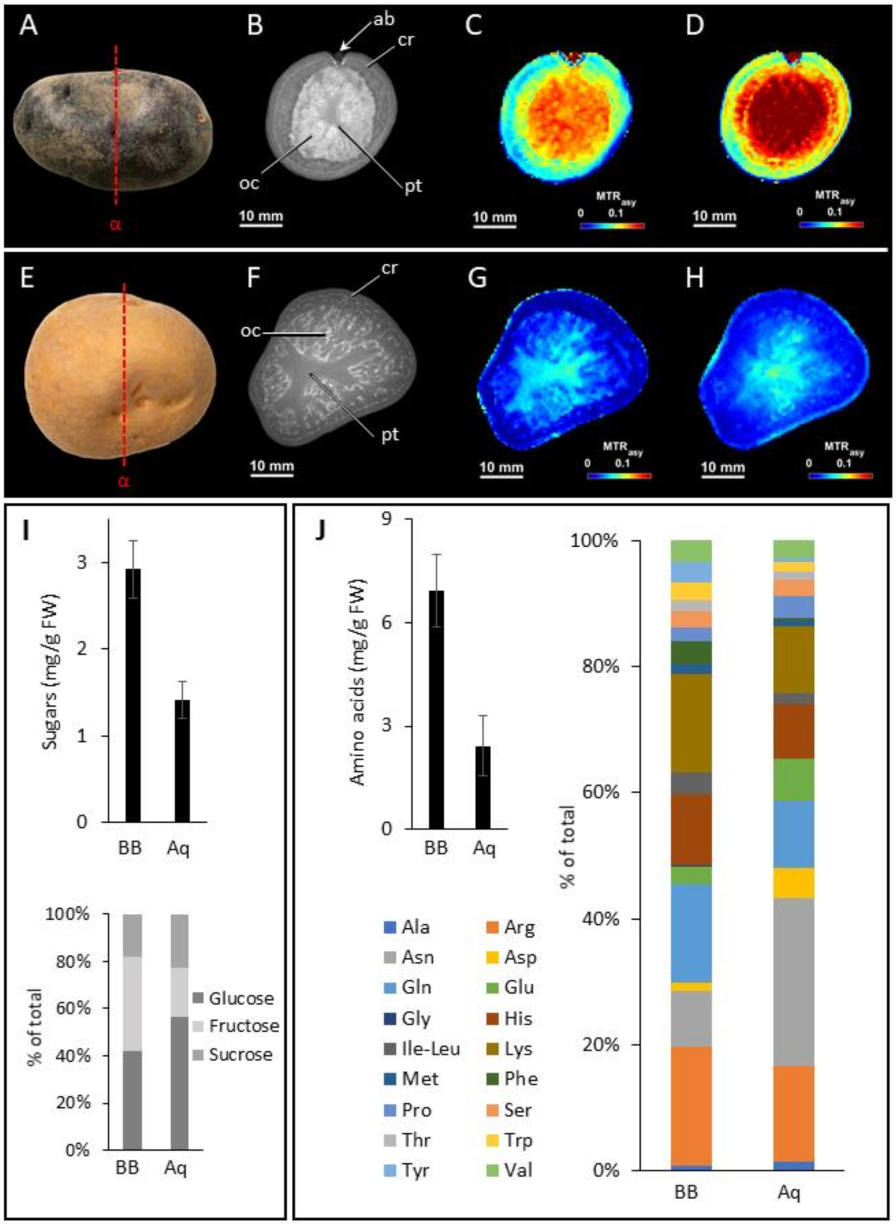


**Supplementary Figure S11. CEST images of potato cultivars and biochemical quantification of sugars and amino acids.**

**(A)** Photographic image of the examined potato tuber of variety "Batenser Blauhörnchen". The grey dashed line indicates the orientation of the slice measured by MR. **(B)** Reference MR image showing the structural arrangement of tissues in 2D. **(C, D)** Visualization of sugar **(C)** and amino acid distribution **(D)** in the same virtual section measured by CEST. **(E-H)** Photographic image of the examined potato tuber of the variety "Aquila" and MRI analysis as described for "Batenser Blauhörnchen". **(I)** Amount and composition of soluble sugars in the variety "Batenser Blauhörnchen" (BB) versus "Aquila" (Aq) measured by ion chromatography (n=3). **(J)** Amount and composition of free amino acids in the variety "Batenser Blauhörnchen" (BB) versus "Aquila" (Aq) measured by mass spectrometry (n=3).

Abbreviations: ab, axillary buds; cr, cortex; oc, outer core; pt, pith.

**Table S1. Measurement times of the CEST experiments.**

| | CEST (incl. reference scan) [h:min:s] | WASSR [h:min:s] |
|---|---|---|
| Fig. 1 / Fig. S1: Young pea | 00:44:00 | 00:40:48 |
| Fig. 1 / Fig. S7: Pea embryo | 01:00:30 | 00:56:06 |
| Fig. 2/3: Barley (dynamic) | 01:12:00 | 01:08:00 |
| Fig. 4: Maize | 00:36:40 | 00:34:00 |
| Fig. 5 / Fig. S9: Kiwi | 00:44:00 | 00:48:48 |
| Fig. 5: Sugar beet | 00:49:30 | 01:30:54 |
| Fig. 5: Sugar cane | 01:17:00 | 01:25:24 |
| Fig. 5: Potato | 01:10:12 | 01:06:18 |
| Fig. S3/S4/S5/S6: Pea | 00:22:00 | 00:08:24 |
| Fig. S8E: Barley (WT) | 00:50:24 | 00:38:16 |
| Fig. S8G: Barley (mutant) | 01:15:36 | 01:13:12 |

The WASSR measurements were always carried out with the same repetition times TR as the corresponding CEST measurements. And a large number of ppm-offsets was measured. Both led to relatively long measurements (usually similar in length to the CEST measurement itself). It should be noted here that a significant acceleration of the WASSR measurement time is possible by reducing TR and the number of ppm-offsets. The CEST measurements themselves can also be accelerated, particularly through shorter recovery times and faster readouts. These time optimizations should be made for 3D measurements at the latest, as mentioned in the discussion section.

**Movie S1.**

Dynamic imaging of sugars and amino acids via CEST within a growing barley caryopsis.

## REFERENCES AND NOTES


1. Y. Yang, M. A. Saand, L. Huang, W. B. Abdelaal, J. Zhang, Y. Wu, J. Li, M. H. Sirohi, F. Wang, Applications of multi-omics technologies for crop improvement. *Front. Plant Sci.* **12**, 563953 (2021).

2. B. P. Sheth, V. S. Thaker, Plant systems biology: Insights, advances and challenges. *Planta* **240**, 33–54 (2014).

3. L. W. Sumner, Z. Lei, B. J. Nikolau, K. Saito, Modern plant metabolomics: Advanced natural product gene discoveries, improved technologies, and future prospects. *Nat. Prod. Rep.* **32**, 212–229 (2015).

4. T. L. Fuss, L. L. Cheng, Metabolic imaging in humans. *Top. Magn. Reson. Imaging* **25**, 223–235 (2016).

5. L. Borisjuk, P. Horn, K. Chapman, P. M. Jakob, A. Gündel, H. Rolletschek, Seeing plants as never before. *New Phytol.*. **238**, 1775–1794 (2023).

6. D. W. McRobbie, E. A. Moore, M. J. Graves, M. R. Prince, *MRI from Picture to Proton: 2nd ed.* (Cambridge Univ. Press, 2007).

7. T. R. Brown, B. M. Kincaid, K. Ugurbil, NMR chemical shift imaging in three dimensions. *Proc. Natl. Acad. Sci. U.S.A.* **79**, 3523–3526 (1982).

8. A. Haase, J. Frahm, W. Hänicke, D. Matthaei, 1H NMR chemical shift selective (CHESS) imaging. *Phys. Med. Biol.* **30**, 341–344 (1985).

9. L. Borisjuk, G. Melkus, “Nuclear magnetic resonance imaging of metabolites in plants and animals” in *In Vivo Imaging: New Research* (Nova Science Publishers, 2014), pp. 1–48.

10. G. Melkus, H. Rolletschek, R. Radchuk, J. Fuchs, T. Rutten, U. Wobus, T. Altmann, P. Jakob, L. Borisjuk, The metabolic role of the legume endosperm: A noninvasive imaging study. *Plant Physiol.* **151**, 1139–1154 (2009).

11. S. M. Glidewell, NMR imaging of developing barley grains. *J. Cereal Sci.* **43**, 70–78 (2006).

12. H. Rolletschek, S. Mayer, B. Boughton, S. Wagner, S. Ortleb, C. Kiel, U. Roessner, L. Borisjuk, The metabolic environment of the developing embryo: A multidisciplinary approach on oilseed rapeseed. *J. Plant Physiol.* **265**, 153505 (2021).

13. C. Juchem, R. A. de Graaf, $B_0$ magnetic field homogeneity and shimming for in vivo magnetic resonance spectroscopy. *Anal. Biochem.* **529**, 17–29 (2017).

14. W. Köckenberger, Functional imaging of plants by magnetic resonance experiments. *Trends Plant Sci.* **6**, 286–292 (2001).

15. H. Van As, T. Scheenen, F. J. Vergeldt, MRI of intact plants. *Photosynth. Res.* **102**, 213–222 (2009).

16. V. Radchuk, Z. M. Belew, A. Gündel, S. Mayer, A. Hilo, G. Hensel, R. Sharma, K. Neumann, S. Ortleb, S. Wagner, A. Muszynska, C. Crocoll, D. Xu, I. Hoffie, J. Kumlehn, J. Fuchs, F. F. Peleke, J. J. Szymanski, H. Rolletschek, H. H. Nour-Eldin, L. Borisjuk, SWEET11b transports both sugar and cytokinin in developing barley grains. *Plant Cell* **35**, 2186–2207 (2023).

17. G. Melkus, H. Rolletschek, J. Fuchs, V. Radchuk, E. Grafahrend-Belau, N. Sreenivasulu, T. Rutten, D. Weier, N. Heinzel, F. Schreiber, T. Altmann, P. M. Jakob, L. Borisjuk, Dynamic $^{13}C/^{1}H$ NMR imaging uncovers sugar allocation in the living seed. *Plant Biotechnol. J.* **9**, 1022–1037 (2011).

18. E. M. Armstrong, E. R. Larson, H. Harper, C. R. Webb, F. Dohleman, Y. Araya, C. Meade, X. Feng, B. Mukoye, M. J. Levin, B. Lacombe, A. Bakirbas, A. A. Cardoso, D. Fleury, A. Gessler, D. Jaiswal, N. Onkokesung, V. S. Pathare, S. S. Phartyal, S. A. Sevanto, I. Wilson, C. S. Grierson, One hundred important questions facing plant science: An international perspective. *New Phytol.* **238**, 470–481 (2023).

19. N. M. Doll, G. C. Ingram, Embryo–endosperm interactions. *Annu. Rev. Plant Biol.* **73**, 293–321 (2022).

20. J. Liu, M.-W. Wu, C.-M. Liu, Cereal endosperms: Development and storage product accumulation. *Annu. Rev. Plant Biol.* **73**, 255–291 (2022).

21. L. Rosado-Souza, R. Yokoyama, U. Sonnewald, A. R. Fernie, Understanding source–sink interactions: Progress in model plants and translational research to crops. *Mol. Plant* **16**, 96–121 (2023).

22. P. C. M. van Zijl, N. N. Yadav, Chemical exchange saturation transfer (CEST): What is in a name and what isn't? *Magn. Reson. Med.* **65**, 927–948 (2011).

23. B. Wu, G. Warnock, M. Zaiss, C. Lin, M. Chen, Z. Zhou, L. Mu, D. Nanz, R. Tuura, G. Delso, An overview of CEST MRI for non-MR physicists. *EJNMMI Phys.* **3**, 19 (2016).

24. R. Podda, D. Delli Castelli, G. Digilio, M. L. Gullino, S. Aime, Asparagine in plums detected by CEST-MRI. *Food Chem.* **169**, 1–4 (2015).

25. G. Pagés, C. Deborde, M. Lemaire-Chamley, A. Moing, J.-M. Bonny, Correction to: MRSI vs CEST MRI to understand tomato metabolism in ripening fruit: Is there a better contrast?. *Anal. Bioanal. Chem.* **413**, 1777 (2021).

26. P. Gao, T. D. Quilichini, H. Yang, Q. Li, K. T. Nilsen, L. Qin, V. Babic, L. Liu, D. Cram, A. Pasha, E. Esteban, J. Condie, C. Sidebottom, Y. Zhang, Y. Huang, W. Zhang, P. Bhowmik, L. V. Kochian, D. Konkin, Y. Wei, N. J. Provart, S. Kagale, M. Smith, N. Patterson, C. S. Gillmor, R. Datla, D. Xiang, Evolutionary divergence in embryo and seed coat development of U's Triangle *Brassica* species illustrated by a spatiotemporal transcriptome atlas. *New Phytol.* **233**, 30–51 (2022).

27. K. M. Ward, A. H. Aletras, R. S. Balaban, A new class of contrast agents for MRI based on proton chemical exchange dependent saturation transfer (CEST). *J. Magn. Reson.* **143**, 79–87 (2000).

28. J.-H. Oh, H.-G. Kim, D.-C. Woo, S. J. Rhee, S. Y. Lee, G.-H. Jahng, Preliminary phantom experiments to map amino acids and neurotransmitters using MRI. *Prog. Med. Phys.* **29**, 29 (2018).

29. T. Meitzel, R. Radchuk, E. L. McAdam, I. Thormählen, R. Feil, M. E. And, A. Hilo, P. Geigenberger, J. J. Ross, J. E. Lunn, L. Borisjuk, Trehalose 6-phosphate promotes seed filling by activating auxin biosynthesis. *New Phytol..* **229**, 1553–1565 (2021).

30. L. Borisjuk, H. Rolletschek, V. Radchuk, "Advances in the understanding of barley plant physiology: factors determining grain development, composition, and chemistry" in *Achieving Sustainable Cultivation of* Barley, G. F. C. Li, Ed. (Burleigh Dodds Science Publishing, 2020), pp. 53–96.

31. D. Rousseau, T. Widiez, S. Tommaso, H. Rositi, J. Adrien, E. Maire, M. Langer, C. Olivier, F. Peyrin, P. Rogowsky, Fast virtual histology using x-ray in-line phase tomography: Application to the 3D anatomy of maize developing seeds. *Plant Methods* **11**, 55(2015).

32. M. Langer, A. Hilo, J.-C. Guan, K. E. Koch, H. Xiao, P. Verboven, A. Gündel, S. Wagner, S. Ortleb, V. Radchuk, S. Mayer, B. Nicolai, L. Borisjuk, H. Rolletschek, Causes and consequences of endogenous hypoxia on growth and metabolism of developing maize kernels. *Plant Physiol.* **192**, 1268–1288 (2023).

33. N. M. Doll, N. Depège-Fargeix, P. M. Rogowsky, T. Widiez, Signaling in early maize kernel development. *Mol. Plant* **10**, 375–388 (2017).

34. Q. Sun, Y. Li, D. Gong, A. Hu, W. Zhong, H. Zhao, Q. Ning, Z. Tan, K. Liang, L. Mu, D. Jackson, Z. Zhang, F. Yang, F. Qiu, A NAC-EXPANSIN module enhances maize kernel size by controlling nucellus elimination. *Nat. Commun.* **13**, 5708 (2022).

35. N. Verbruggen, C. Hermans, Proline accumulation in plants: A review. *Amino Acids* **35**, 753–759 (2008).

36. D. P. Richardson, J. Ansell, L. N. Drummond, The nutritional and health attributes of kiwifruit: A review. *Eur. J. Nutr.* **57**, 2659–2676 (2018).

37. J. M. McGrath, L. Panella, "Sugar beet breeding" in *Plant Breeding* Reviews (Wiley & Sons Inc., 2018), pp. 167–218.

38. U. G. Mueller-Lisse, S. Murer, U. L. Mueller-Lisse, M. Kuhn, J. Scheidler, M. Scherr, Everyman's prostate phantom: Kiwi-fruit substitute for human prostates at magnetic resonance imaging, diffusion-weighted imaging and magnetic resonance spectroscopy. *Eur. Radiol.* **27**, 3362–3371 (2017).

39. G. Hajjar, S. Quellec, S. Challois, L. Bousset-Vaslin, G. Joly, C. Langrume, C. Deleu, L. Leport, M. Musse, Characterization of the water shortage effects on potato tuber tissues during growth using MRI relaxometry and biochemical parameters. *Plants* **11** 1918 (2022).

40. G. Hajjar, S. Quellec, J. Pépin, S. Challois, G. Joly, C. Deleu, L. Leport, M. Musse, MRI investigation of internal defects in potato tubers with particular attention to rust spots induced by water stress. *Postharvest Biol. Technol.* **180** 111600 (2021).

41. S. Murer, J. Scheidler, U. L. Mueller-Lisse, M. Helling, M. Scherr, U. G. Mueller-Lisse, Two-centre comparative experimental study of biparametric MRI at 3.0 T with and without endorectal coil using kiwifruit (*Actinidia deliciosa*) as a phantom for human prostate. *Eur. Radiol. Exp.* **3**, 30 (2019).

42. L. Zhang, Z. Tang, H. Zheng, C. Zhong, Q. Zhang, Comprehensive analysis of metabolome and transcriptome in fruits and roots of kiwifruit. *Int. J. Mol. Sci.* **24** 1299 (2023).

43. M. C. Elliott, G. D. Weston, "Biology and physiology of the sugar-beet plant" in *The Sugar Beet Crop* (Springer, 1993), pp. 37–66.

44. P. Fasahat, M. Aghaeezadeh, L. Jabbari, S. Sadeghzadeh Hemayati, P. Townson, Sucrose accumulation in sugar beet: From fodder beet selection to genomic selection. *Sugar Tech* **20**, 635–644 (2018).

45. A. Jammer, A. Albacete, B. Schulz, W. Koch, F. Weltmeier, E. van der Graaff, H. W. Pfeifhofer, T. G. Roitsch, Early-stage sugar beet taproot development is characterized by three distinct physiological phases. *Plant Direct* **4**, e00221 (2020).

46. R. Metzner, D. van Dusschoten, J. Bühler, U. Schurr, S. Jahnke, Belowground plant development measured with magnetic resonance imaging (MRI): Exploiting the potential for non-invasive trait quantification using sugar beet as a proxy. *Front. Plant Sci.* **5**, 469(2014).

47. F. C. Botha, G. Scalia, A. Marquardt, K. Wathen-Dunn, Sink strength during sugarcane culm growth: Size matters. *Sugar Tech* **25**, 1047–1060 (2023).

48. J. M. García, C. Molina, R. Simister, C. B. Taibo, L. Setten, L. E. Erazzú, L. D. Gómez, A. Acevedo, Chemical and histological characterization of internodes of sugarcane and energy-cane hybrids throughout plant development. *Ind. Crops Prod.* **199** 116739 (2023).

49. D. A. Ferreira, M. C. M. Martins, A. Cheavegatti-Gianotto, M. S. Carneiro, R. R. Amadeu, J. A. Aricetti, L. D. Wolf, H. P. Hoffmann, L. G. F. de Abreu, C. Caldana, Metabolite profiles of sugarcane culm reveal the relationship among metabolism and axillary bud outgrowth in genetically related sugarcane commercial cultivars. *Front. Plant Sci.* **9**, 857 (2018).

50. M. Sadoine, Y. Ishikawa, T. J. Kleist, M. M. Wudick, M. Nakamura, G. Grossmann, W. B. Frommer, C. H. Ho, Designs, applications, and limitations of genetically encoded fluorescent sensors to explore plant biology. *Plant Physiol.* **187**, 485–503 (2021).

51. A. Walia, R. Waadt, A. M. Jones, Genetically encoded biosensors in plants: Pathways to discovery. *Annu. Rev. Plant Biol.* **69**, 497–524 (2018).

52. M. Zaiss, G. Angelovski, E. Demetriou, M. T. McMahon, X. Golay, K. Scheffler, QUESP and QUEST revisited – Fast and accurate quantitative CEST experiments. *Magn. Reson. Med.* **79**, 1708–1721 (2018).

53. S. Aime, L. Calabi, L. Biondi, M. De Miranda, S. Ghelli, L. Paleari, C. Rebaudengo, E. Terreno, Iopamidol: Exploring the potential use of a well-established x-ray contrast agent for MRI. *Magn. Reson. Med.* **53**, 830–834 (2005).

54. V. Radchuk, L. Borisjuk, R. Radchuk, H.-H. Steinbiss, H. Rolletschek, S. Broeders, U. Wobus, Jekyll encodes a novel protein involved in the sexual reproduction of barley. *Plant Cell* **18**, 1652–1666 (2006).

55. A. Guendel, H. Rolletschek, S. Wagner, A. Muszynska, L. Borisjuk, Micro imaging displays the sucrose landscape within and along its allocation pathways. *Plant Physiol.* **178**, 1448–1460 (2018).

56. V. Radchuk, V. Tran, A. Hilo, A. Muszynska, A. Gündel, S. Wagner, J. Fuchs, G. Hensel, S. Ortleb, E. Munz, H. Rolletschek, L. Borisjuk, Grain filling in barley relies on developmentally controlled programmed cell death. *Commun. Biol.* **4**, 428 (2021).

57. H. Rolletschek, M.-R. Hajirezaei, U. Wobus, H. Weber, Antisense-inhibition of ADP-glucose pyrophosphorylase in *Vicia narbonensis* seeds increases soluble sugars and leads to higher water and nitrogen uptake. *Planta* **214**, 954–964 (2002).

58. J. Trevisan, P. P. Angelov, A. D. Scott, P. L. Carmichael, F. L. Martin, IRootLab: A free and open-source MATLAB toolbox for vibrational biospectroscopy data analysis. *Bioinformatics* **29**, 1095–1097 (2013).

59. M. Kim, J. Gillen, B. A. Landman, J. Zhou, P. C. M. van Zijl, Water saturation shift referencing (WASSR) for chemical exchange saturation transfer (CEST) experiments. *Magn. Reson. Med.* **61**, 1441–1450 (2009).

60. G. Varma, R. E. Lenkinski, E. Vinogradov, Keyhole chemical exchange saturation transfer. *Magn. Reson. Med.* **68**, 1228–1233 (2012).

61. I. Tkáč, Z. Starčuk, I. Y. Choi, R. Gruetter, In vivo $^{1}$H NMR spectroscopy of rat brain at 1 ms echo time. *Magn. Reson. Med.* **41**, 649–656 (1999).

62. M. Zaiß, B. Schmitt, P. Bachert, Quantitative separation of CEST effect from magnetization transfer and spillover effects by Lorentzian-line-fit analysis of z-spectra. *J. Magn. Reson.* **211**, 149–155 (2011).

63. M. Zaiss, J. Xu, S. Goerke, I. S. Khan, R. J. Singer, J. C. Gore, D. F. Gochberg, P. Bachert, Inverse *Z*-spectrum analysis for spillover-, MT-, and $T_1$-corrected steady-state pulsed CEST-MRI - application to pH-weighted MRI of acute stroke. *NMR Biomed.* **27**, 240–252 (2014).

64. D. E. Woessner, S. Zhang, M. E. Merritt, A. D. Sherry, Numerical solution of the Bloch equations provides insights into the optimum design of PARACEST agents for MRI. *Magn. Reson. Med.* **53**, 790–799 (2005).

65. J. Zhou, J.-F. Payen, D. A. Wilson, R. J. Traystman, P. C. M. van Zijl, Using the amide proton signals of intracellular proteins and peptides to detect pH effects in MRI. *Nat. Med.* **9**, 1085–1090 (2003).

66. R. Pohmann, M. von Kienlin, Accurate phosphorus metabolite images of the human heart by 3D acquisition-weighted CSI. *Magn. Reson. Med.* **45**, 817–826 (2001).

67. P. T. Callaghan, “NMR microscopy” in *Encyclopedia of Spectroscopy and Spectrometry* (Academic Press, 2016), pp. 154–163.

68. B. A. Jung, M. Weigel, Spin echo magnetic resonance imaging. *J. Magn. Reson. Imaging* **37**, 805–817 (2013).

69. M. A. Bernstein, S. B. Fain, S. J. Riederer, Effect of windowing and zero-filled reconstruction of MRI data on spatial resolution and acquisition strategy. *J. Magn. Reson. Imaging* **14**, 270–280 (2001).
